# A Review of Electrified Methane Conversion: Utilizing Electrocatalysis, Plasma, Electric‐Field and Electro‐Thermal Technologies

**DOI:** 10.1002/tcr.202400256

**Published:** 2025-06-23

**Authors:** Alen Rupnik, Igor Shlyapnikov, Miha Grilc, Gleb Veryasov, David Bajec, Blaž Likozar

**Affiliations:** ^1^ Department of Catalysis and Chemical Reaction Engineering National Institute of Chemistry Hajdrihova 19 1000 Ljubljana Slovenia; ^2^ Centre of Excellence Low‐Carbon Technologies Hajdrihova 19 1000 Ljubljana Slovenia; ^3^ University of Nova Gorica Vipavska 13 5000 Nova Gorica Slovenia; ^4^ TotalEnergies OneTech Belgium Zone Industrielle C 7181 Feluy Belgium

**Keywords:** electrification, electrocatalysis, induction heating, methane activation, methane valorisation

## Abstract

Methane, a potent greenhouse gas and a major component of natural gas, holds immense potential as a feedstock for producing value‐added chemicals and fuels. This review examines recent advancements in electrified methane conversion technologies, emphasizing sustainable approaches to mitigate emissions while enabling efficient utilization. The paper explores key methods, including electrocatalysis, plasma‐driven reactions, and electrothermal processes, which leverage renewable electricity to activate methane under mild conditions. Special focus is given to catalyst design, reactor configurations, and process integration, highlighting improvements in selectivity, energy efficiency, and scalability. These technologies offer promising pathways to decarbonize industrial processes and transition toward a circular economy, aligning with global climate and energy goals. By addressing current challenges and identifying future research directions, this review aims to advance the field of methane valorization and support the development of greener chemical manufacturing strategies.

## Graphical abstract

Four predominant technologies for electrified methane conversion.

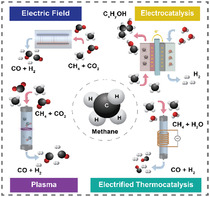

## 1 Introduction

### 1.1 Background

#### 1.1.1 Natural Gas Emissions

**Figure 1:**
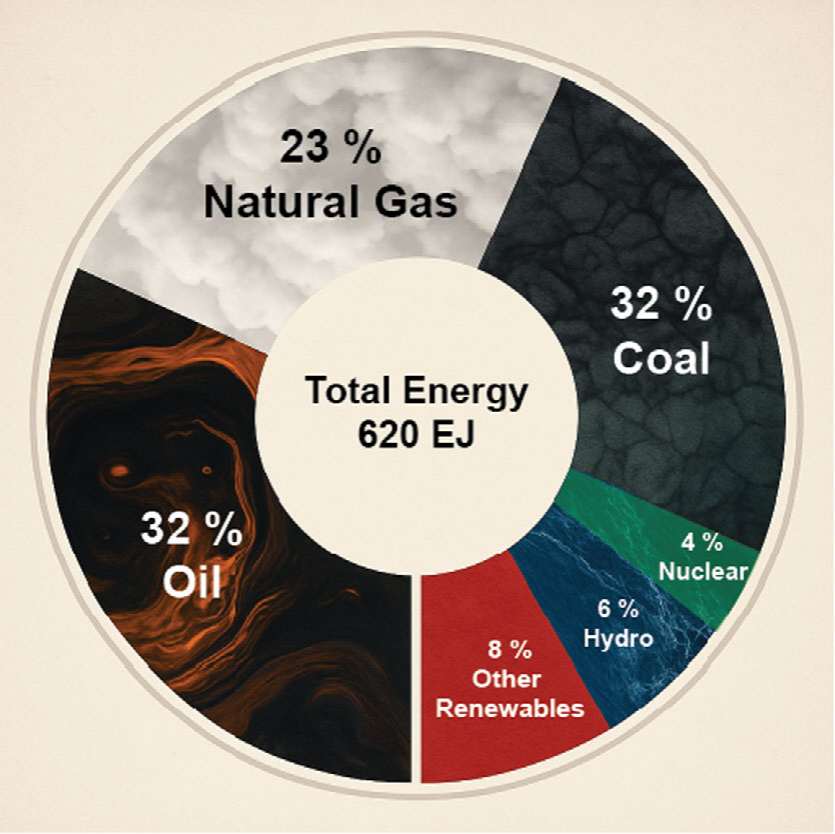
World energy consumption in 2023 by energy source. Reproduced with permission.^[^ ^1^ ^]^ Copyright 2023, International Energy Institute.

Natural gas is a significant global energy source, accounting for nearly 23% of the world's energy consumption (see **Figure** **1**).^[^ ^1^ ^]^ Its relatively low carbon intensity compared to other fossil fuels, such as coal and oil, positions it as a viable and cleaner alternative. This potential is further amplified by the vast natural gas reserves and emerging technologies for its clean conversion, including the production of hydrogen—a promising zero‐carbon fuel for the future. Methane hydrate, often referred to as solidified natural gas (SNG), has lately been a topic of interest, as it can greatly expand the possibilities for methane utilization. These hydrates represent a vast and largely untapped source of methane, opening nearly limitless opportunities for innovative conversion pathways, provided we can access them, positioning methane hydrate as a potential renewable energy source in the long run.^[^ ^2^, ^3^ ^]^

Clean energy production remains a globally recognized priority as the developed world progresses toward achieving net‐zero emissions. This transition has driven the development of numerous renewable energy sources and advanced production technologies. The gradual decline of the fossil fuel era is evident, underscored by initiatives such as the European Union's proposed ban on the production of internal combustion engine vehicles by 2035. The primary impetus for such measures lies in reducing carbon dioxide (CO_2_) emissions, the leading contributor to the greenhouse gas (GHG) effect. However, fossil fuels contribute to GHG emissions not only through combustion but also through the release of methane—the second largest GHG contributor. Methane, the principal component of natural gas, also emerges as a significant waste product from fossil fuel exploration and production.

According to the International Energy Agency (IEA), addressing methane emissions from fossil fuel operations represents one of the most effective near‐term strategies to mitigate climate change. In light of this, a pathway to achieve a 75% reduction in methane emissions from fossil fuel operations by 2030 has been proposed.^[^ ^4^ ^]^

**Figure 2:**
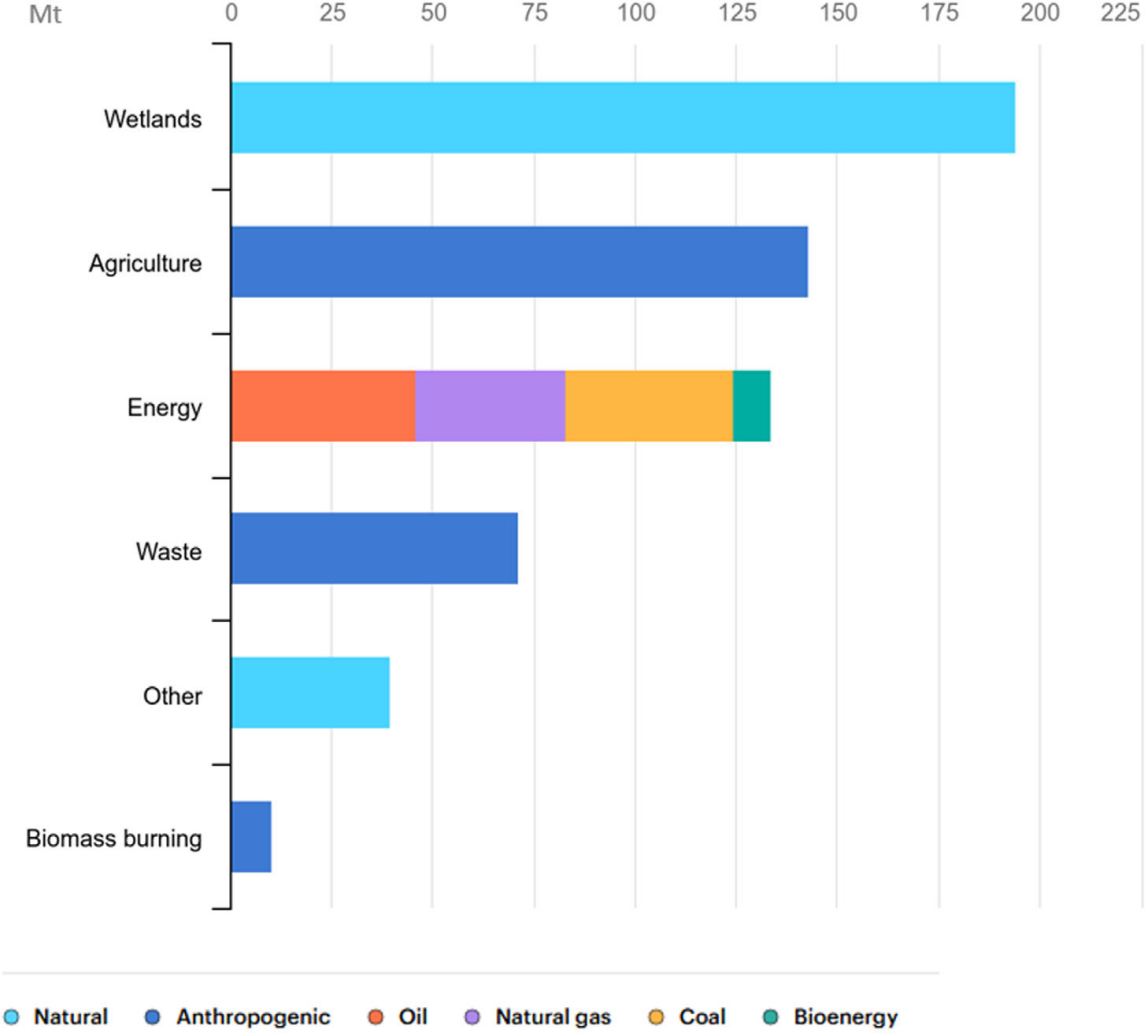
Sources of methane emission for 2023 in Mt of Methane. Reproduced with permission.^[^ ^6^ ^]^ Copyright IEA 2023, Global Methane Tracker 2023, https://www.iea.org/reports/global‐methane‐tracker‐2023, License: CC BY 4.0.

Methane concentrations in the atmosphere are currently around 2.5 times higher than pre‐industrial levels and continue to rise steadily. Comprehensive estimates for 2023 from the Global Methane Budget (**Figure** **2**),^[^ ^5^ ^]^ indicate total methane emissions of ≈570 Mt. These emissions are divided between natural sources (around 40%) and human activities (around 60%).^[^ ^4^, ^6^ ^]^

Given these figures, it is imperative to address all methane emissions arising from human activity. Fossil fuel operations, as a significant source, warrant focused efforts to mitigate their environmental impact. Such measures are essential not only for combating climate change but also for ensuring a sustainable transition to clean energy sources.

**Figure 3:**
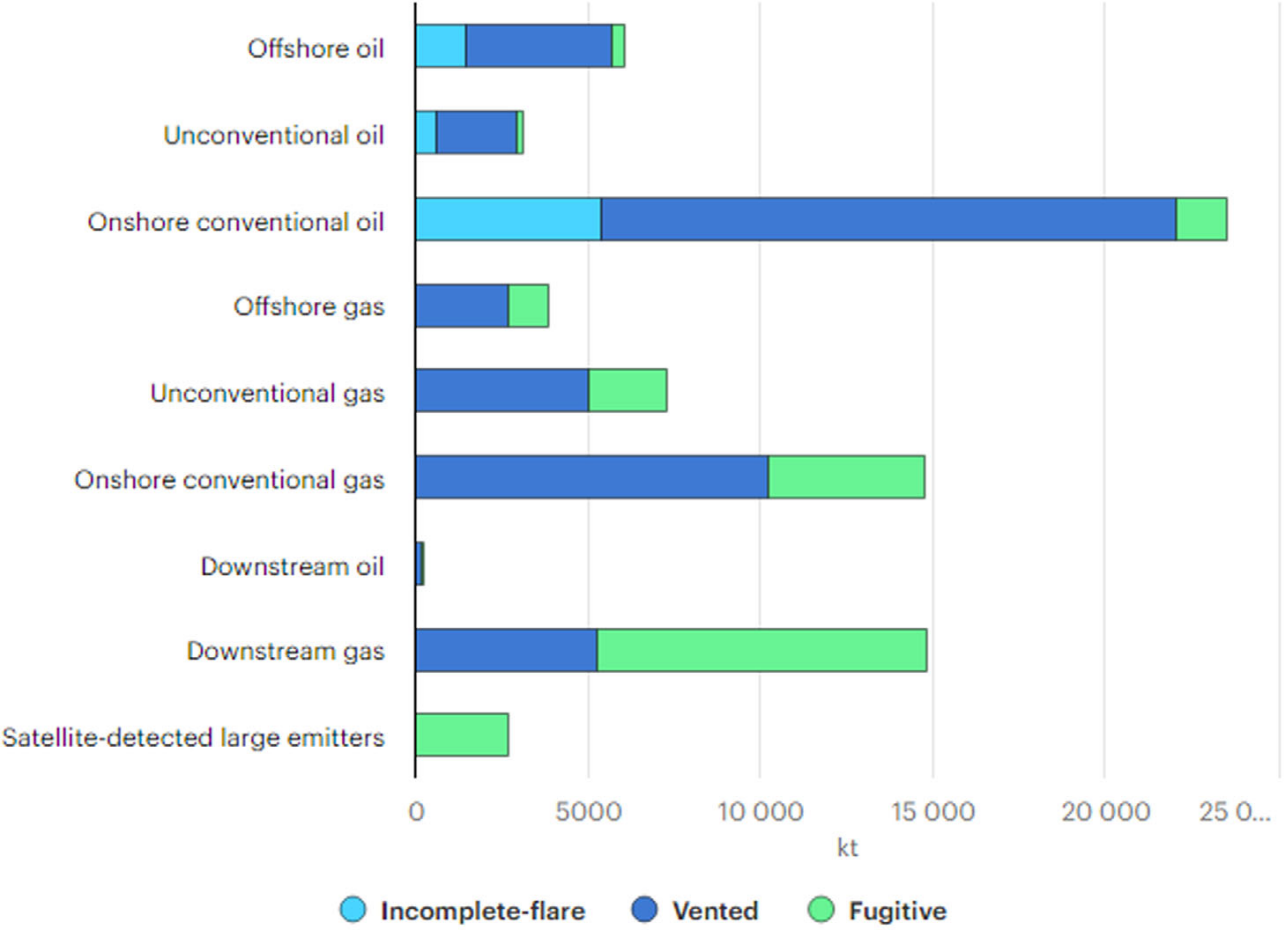
Global Methane Emissions from Fossil Fuel Operations, IEA estimate for 2020. Reproduced with permission.^[^^ 86 ^^]^ Copyright IEA 2020, Methane Tracker Database, https://www.iea.org/data‐and‐statistics/data‐product/methane‐tracker‐database, License: CC BY 4.0.

Methane does not pose a significant environmental problem when it is effectively captured and converted into higher value‐added products such as methanol, ethanol, and other chemicals, not intended to be used as fuels. However, there are substantial opportunities to mitigate methane emissions, particularly in fossil fuel operations. As illustrated in **Figure** **3**, implementing strategies such as methane capture, emissions monitoring, equipment restoration, and on‐site conversion technologies could result in a reduction of ≈54,611 kilotons (72%) of methane emissions from these sources alone.^[^ ^7^ ^]^ These measures represent a critical pathway for both reducing GHG emissions and enhancing the sustainable utilization of methane.

Methane has a relatively short atmospheric lifetime of about a decade, during which it reacts with hydroxyl radicals to form carbon dioxide—a compound that can persist in the atmosphere for centuries. While its lifespan in the atmosphere is brief compared to CO_2_, methane's significant environmental impact arises from its potent heat‐trapping ability. Methane absorbs heat in the Earth's atmosphere ≈80 times more effectively than a CO_2_ molecule over a 20‐year period, making it a critical target for climate mitigation efforts.^[^ ^8^ ^]^

#### 1.1.2 Natural Gas Reserves

**Figure 4:**
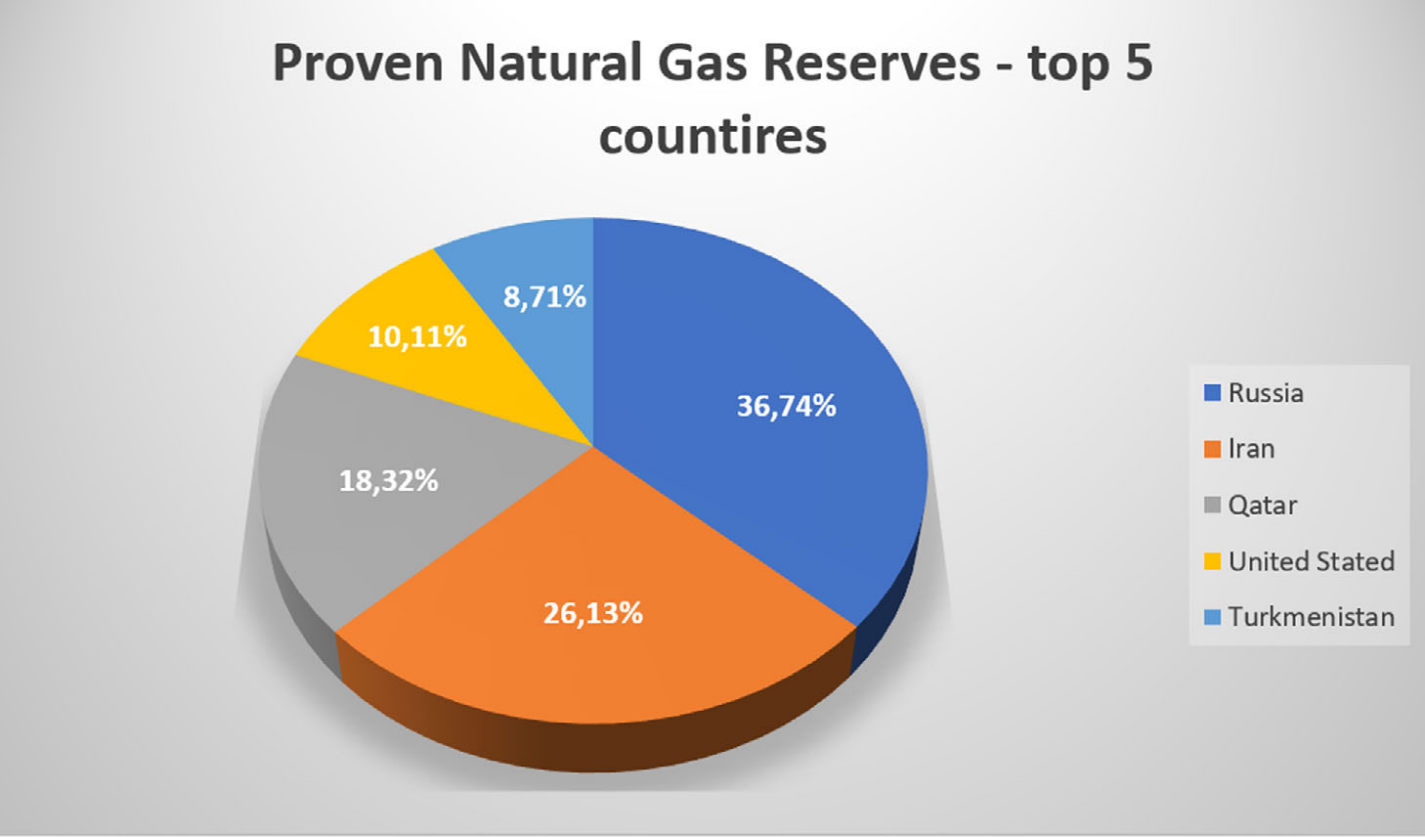
Top 5 Countries with the Largest Natural Gas Reserves. Reproduced with permission.^[^^ 87 ^^]^ Copyright 2025, Worldometer.

Methane reserves exist in various forms and sources, with natural gas reserves representing the largest and most significant contributor. Recent discoveries of substantial shale gas reserves have further expanded the availability of methane, leading to a noticeable decrease in its market price. As shown in **Figure** **4**, Russia holds the largest natural gas reserves globally, followed by Iran and Qatar, highlighting the geographical concentration of this critical resource. These reserves play a pivotal role in meeting global energy demands and shaping the economics of methane utilization.

**Figure 5:**
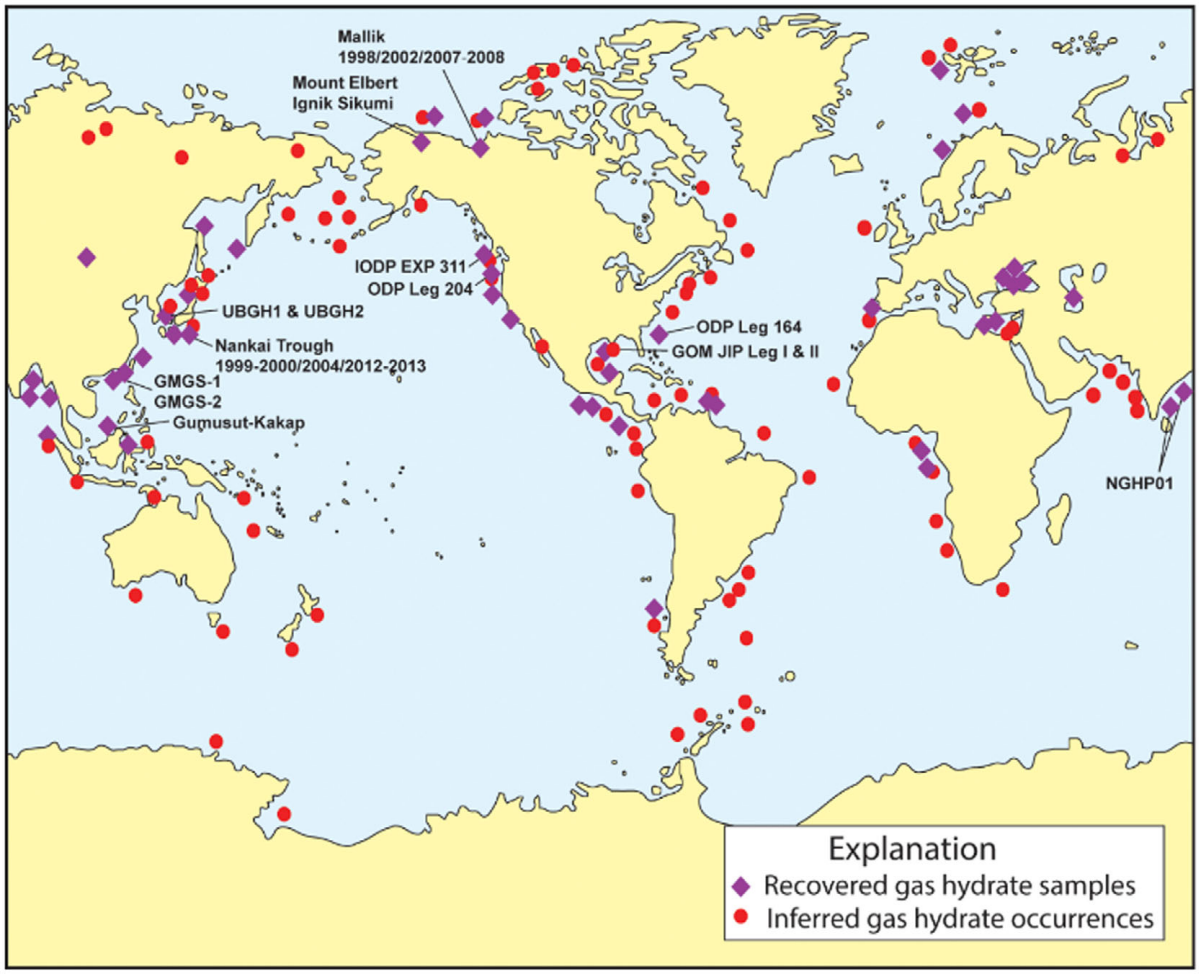
Locations of sampled and inferred methane hydrate occurrences in oceanic sediment. Reproduced with permission.^[^ ^3^ ^]^ Copyright 2025, American Chemical Society.

Recent studies have highlighted the potential utilization of before mentioned methane hydrates, which are found beneath the sea surface, primarily within oceanic sediments (**Figure** **5**). These deposits represent an enormous yet largely untapped source of methane. However, before harnessing methane from hydrates, it is essential to develop green and economically viable solutions for the utilization of methane from existing natural gas reserves. Extracting methane from hydrates poses significant technical challenges, as many deposits are located in deep sediments, often beyond 500 meters below the ocean floor, making access and recovery particularly complex and resource intensive.

### 1.2 Methane Activation

Methane activation and conversion processes, along with coupling reactions, provide pathways for its utilization, but reducing the energy demands of these reactions is essential for improving their environmental footprint. Methane, as the hydrocarbon with the highest hydrogen‐to‐carbon ratio, possesses four strong and highly localized C—H bonds, making it chemically inert in terms of its resistance to bond activation (434 kJ mol^−1^) under mild conditions, as methane also has low polarizability, and lack of functional groups that could facilitate adsorption or reaction at catalytic interfaces. Although the molecule can be activated, a significant challenge arises from the lower chemical and thermodynamic stability of the desired products. Traditionally, most methane is burned for heating and electricity generation, with a smaller fraction used as a vehicle fuel.^[^ ^9^ ^]^

Currently, industrial methane utilization predominantly relies on indirect conversion pathways, most notably steam methane reforming (SMR), partial oxidation of methane (POM), and autothermal reforming (ATR). SMR, the most widely used process, involves the reaction of methane with steam at 700–1100 °C over nickel‐based catalysts to produce syngas (CO and H_2_), but it is highly endothermic and energy intensive, contributing significantly to CO_2_ emissions and suffering from catalyst deactivation due to sintering and coking. POM, a faster and mildly exothermic route, reacts methane with oxygen to yield syngas but requires strict oxygen control to prevent total combustion and suffers from lower selectivity. ATR integrates SMR and POM in a single reactor to balance thermal requirements, yet it faces challenges in reactor complexity and precise reactant management. Across all these methods, common limitations persist: high operational temperatures, suboptimal carbon efficiency due to CO_2_ byproducts, poor adaptability for small‐scale or remote applications, and catalyst degradation under prolonged exposure. Furthermore, these processes do not offer direct routes to high‐value chemicals such as methanol or olefins, with overoxidation and low selectivity hindering direct methane functionalization.^[^ ^10^, ^11^ ^]^ Syngas does serve as a versatile precursor for synthesizing a wide range of hydrocarbons and alcohols using catalytic processes powered by various energy sources,^[^ ^12^, ^13^ ^]^ the most well‐known such process is Fischer–Tropsch process. Products like methanol, ethanol, and other higher hydrocarbons are more reactive than methane and thermodynamically favorable. However, slow reaction kinetics often hinder the cost efficiency of methanol production, prompting the exploration of new direct conversion and activation methods. These limitations underscore the urgent need for novel, renewable and energy‐efficient pathways. Recent studies focus on discovering innovative catalysts and employing alternative power sources and reactor designs to exploit methane resources more efficiently and sustainably, preferably onsite.

A growing interest in non‐oxidative methane conversion methods is evident, particularly because these processes avoid the formation of CO_2_.^[^ ^14^ ^]^ In oxygen‐free environments, methane can be directly converted into higher hydrocarbons while releasing hydrogen, ensuring complete utilization of its carbon and hydrogen atoms. Non‐oxidative activation methods include electrocatalysis, electric field‐mediated conversion, magnetic field and plasma‐powered activation, halogenation, and thermal dry reforming, but these processes are yet to be widely commercialized.

**Figure 6:**
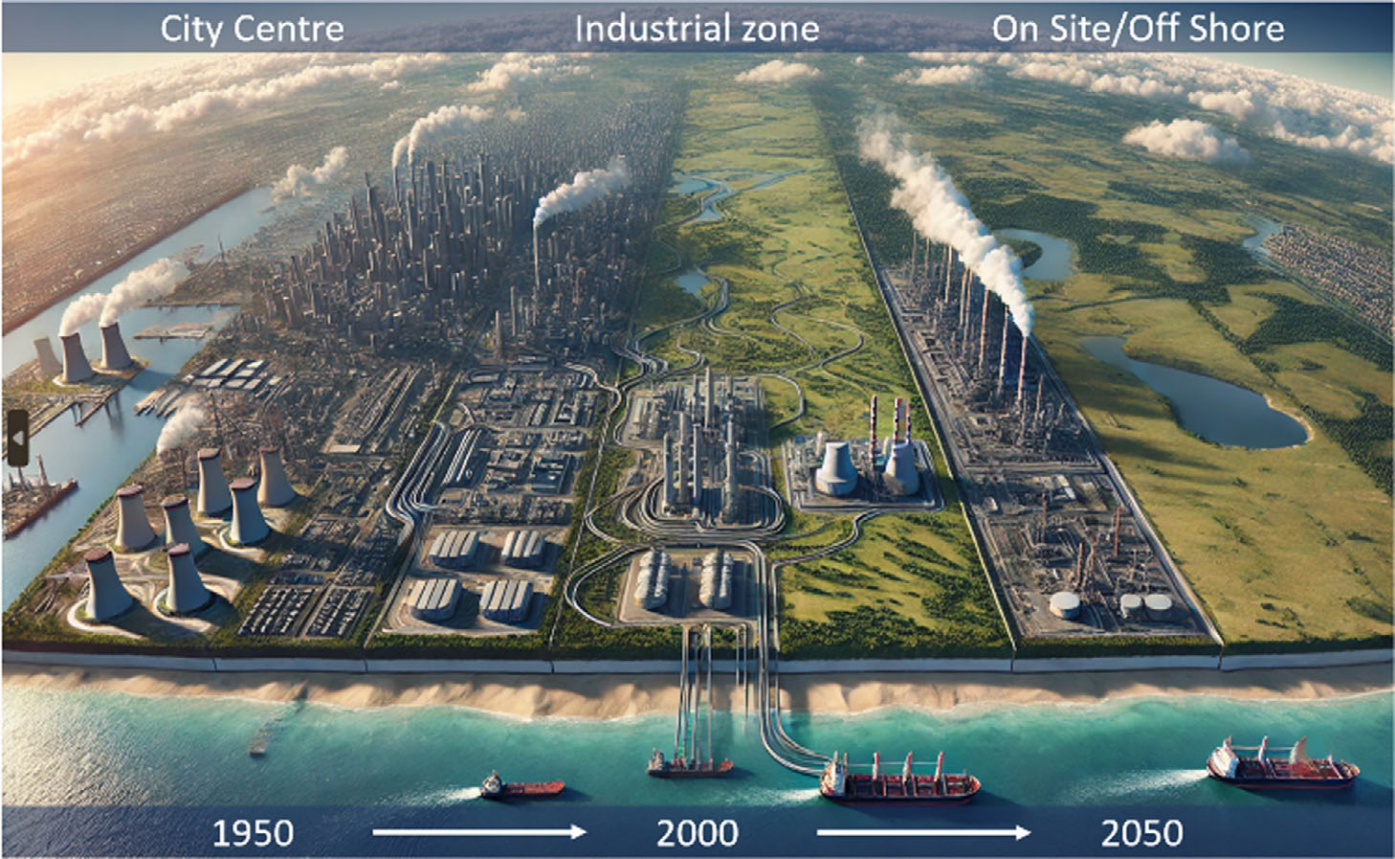
Industry shift from city centers in 1950s to city adjacent industrial zones nowadays, to on‐site/off shore plants in the future.

The primary objective of this publication is to provide readers with a comprehensive understanding of innovative methodologies and reactor designs for efficient methane conversion, aiming for near‐zero GHG emissions. To mitigate these transport‐related emissions, we propose a paradigm shift toward deploying new technologies directly at methane sources, facilitating on‐site/offshore conversion as presented in **Figure** **6**.

**Figure 7:**
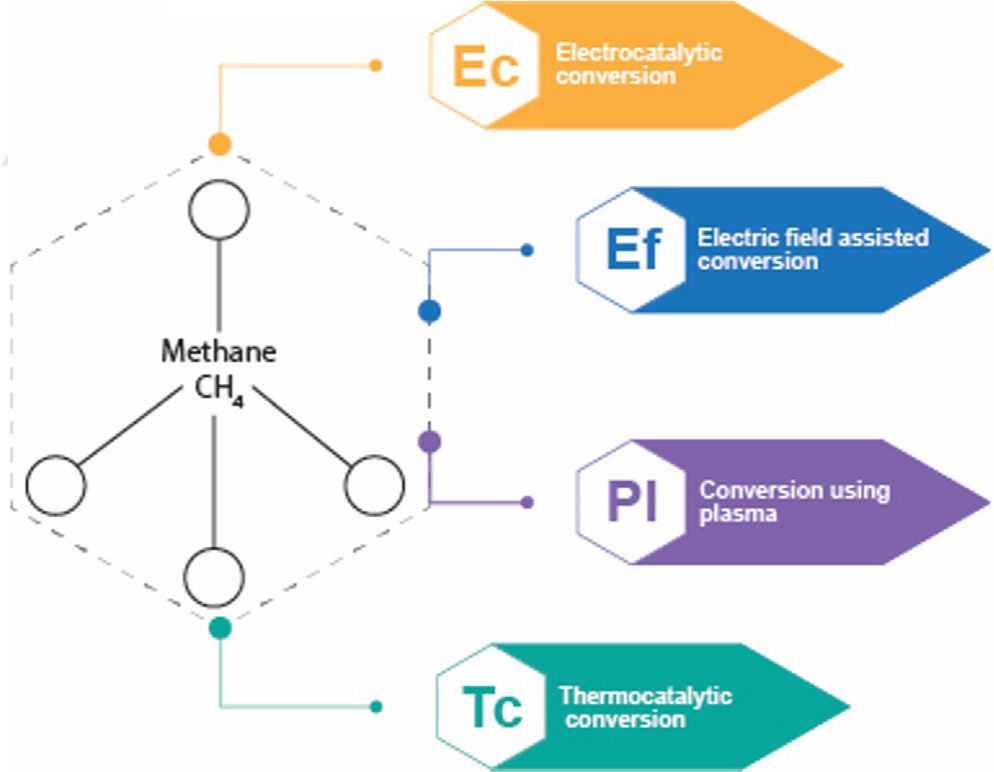
Four main bespoke methane conversions using electricity.

In addition, this publication will analyze the most practical and valuable final products derived from methane conversion, offering insights into their applications. The four principal methods for methane conversion utilizing electricity as an energy source will also be explored, highlighting their mechanisms, efficiencies, and environmental benefits. This integrated approach aims to advance the sustainable utilization of methane while aligning with global climate and energy goals. (**Figure** **7**).

## 2 Electrochemical Conversion of Methane

### 2.1 Electrocatalytic Conversion

Electrocatalytic methane conversion presents a compelling pathway for the valorization of natural gas using electricity, particularly from renewable sources, under ambient or near‐ambient conditions. Unlike thermal or oxidative methods that rely on high temperatures and often yield CO_2_ as a byproduct, electrochemical approaches offer the potential for selective, low‐emission conversion of methane to oxygenates, alcohols, and hydrocarbons through precisely tuned redox environments at the electrode–electrolyte interface.

At the core of electrocatalytic activation lies the multielectron, proton‐coupled oxidation of methane, where controlling the number and timing of electron transfers is critical to avoid overoxidation and to favor partial functionalization. This contrasts sharply with traditional catalysis, as the electrochemical potential can serve as an external, non‐thermal driving force to selectively increase concentration of wanted intermediates and lower activation barriers. However, the low polarity and electron affinity of CH_4_ result in poor adsorption and electron transfer kinetics, making the first C—H bond activation step rate‐limiting in most systems. Overcoming these hurdles through electrocatalysis under ambient or moderate conditions has become a focal point in catalytic research, offering the potential for selective, energy‐efficient chemical production while leveraging renewable electricity. Representative methods for electrocatalytic methane conversion are presented in **Table** **1**.

**Table 1:** Representative studies on electrocatalytic conversion of methane.

Means of conversion	Anode/Catalysts	Reactor/electrolyzer type	Electrolysis condition	CH_4_ Conversion/rate	FE [%]	Primary Products	References
Oxidation	Cu‐complex (2.5%) on Carbon Vulcan	SEMR‐FC	0 V vs RRDE; 0,11 mA cm^−2^	1.85 mol L^−1^ h^−1^	–	Methanol, H_2_O	[28]
	Pd_(90)_Zn_(10)_/C	Proton‐exchange membrane fuel cell (PEMFC)	80 °C; 3,51 mA cm^−2^; 0,15 V	–	–	CO_2_, H_2_O	[23]
	Ni‐BZCYYb	SOFC	500 °C; 0,37 Wcm^−2^; 0,75 V	3%	–	H_2_, CO, Electricity	[26]
	NiO/NiHF	3 electrode cell	25 °C; 1,4 V vs RHE; 0,04 mA cm^−2^	–	89% EtOH 54% MeOH	Methanol, Ethanol	[15]
	NiO/Ni	3 electrode cell	25 °C; 1,4 V vs RHE; 3 mA cm^−2^	–	89% EtOH 17% MeOH	Methanol, Ethanol	[16]
	Pt wire	High pressure – 3 electrode cell	130 °C; 675 psi; 0,91 mA	TOF MeOH 0,32 h^−1^	–	Methanol, Methane diol, Formic acid	[21]
	Pt anode/Ti^IV^ in H_2_SO_4_	3 electrode cell	25 °C; 2,3 V vs Hg_2_SO_4_/Hg	TOF 64 h^−1^	65,8%	Methyl sulphate	[17]
	Pt anode/V^V^ in H_2_SO_4_	3 electrode cell	25 °C; 2,26 V vs Hg_2_SO_4_/Hg	TOF 483 h^−1^	63,5%	Methyl sulphate	[17]
	Pt anode/Cr^VI^ in H_2_SO_4_	3 electrode cell	25 °C; 2,17 V vs Hg_2_SO_4_/Hg	TOF 107 h^−1^	57,8%	Methyl sulphate	[17]
							
OCM	Co_3_O_4_/ZrO_2_ NT	3 electrode cell	25 °C; 1,6 V vs. RHE; 1 mA cm^−2^	2416 μmol g_cat_ −1 h^−1^	–	1‐propanol, 2‐propanol, Ethanol	[20]
	Co_3_O_4_/ZrO_2_	2 electrode cell	25 °C; 2 V vs. Pt; 8,7 mA cm^−2^	40%	–	1‐propanol, 2‐propanol, acetaldehyde	[19]
	SrFeMoO	SOE	850 °C; −0,8 to −0,6 V vs. oxygen reference electrode	–	70% Ethylene	Ethylene	[24]
	0.075Fe–Sr2Fe1.5Mo0.5O6−δ	SOE	850 °C; 1,6 V	41%	–	Ethylene, ethane	[25]
							
Partial oxidation	Pt/C Basf	AAEMFC	25 °C; 0,3 V	20%	–	Methanol	[27]
	Pd_(90)_Cu_(10)_/C	PEMFC	25 °C; 0,3 V vs. Ag/AgCl	13 mol L^−1^ h^−1^	–	methanol, dimethyl ether, methyl and K formate	[22]
	ZrO_2_:NiCo_2_O_4_	2 electrode cell	25 °C; 2,0 V; 2,3 mA cm^−2^	47,5%	–	Propionic acid, Acetic acid and Acetone	[18]

Catalyst design lies at the heart of advancements in methane electrocatalysis. Among the notable breakthroughs, Ni‐based catalysts have emerged as powerful tools for methane activation. For instance, the NiO/Ni hollow fiber electrode has demonstrated exceptional performance in producing ethanol through electrocatalytic oxidation. By engineering the NiO/Ni interface, researchers achieved a remarkable Faradaic efficiency (FE) of 89% at 1.4 V vs. reversible hydrogen electrode (RHE), with a yield of 25 μmol g^−1^ h^−1^. This success is attributed to the unique ability of the NiO/Ni interface to facilitate both C—H activation and C—C coupling, which are critical steps in ethanol synthesis. This system represents a step forward in addressing the inherent challenges of methane activation under mild conditions.^[^ ^15^, ^16^ ^]^ The FE of electrocatalytic systems critically determines their viability for industrial applications. For example, high FE values were achieved by Deng et al. in an electrolysis cell under ambient conditions using d^0^ early transition metals such as titanium (IV), vanadium (V), and chromium (VI). These metals were employed as homogeneous molecular eletrocatalysts in 98% sulfuric acid, activating methane through a turnover‐limiting one‐electron oxidation mechanism. The primary product of the reaction was methyl bisulfate (CH_3_OSO_3_H), formed with a high FE of up to 84.5% under 3 bar CH_4_ at room temperature. The study highlights the catalytic stability, achieving turnover numbers exceeding 45 000 over 72 h without significant catalyst degradation. This work establishes the general applicability of d^0^ early transition metals for methane activation, offering a pathway for efficient, ambient, and selective conversion of methane into value‐added chemicals.^[^ ^17^ ^]^

**Figure 8:**
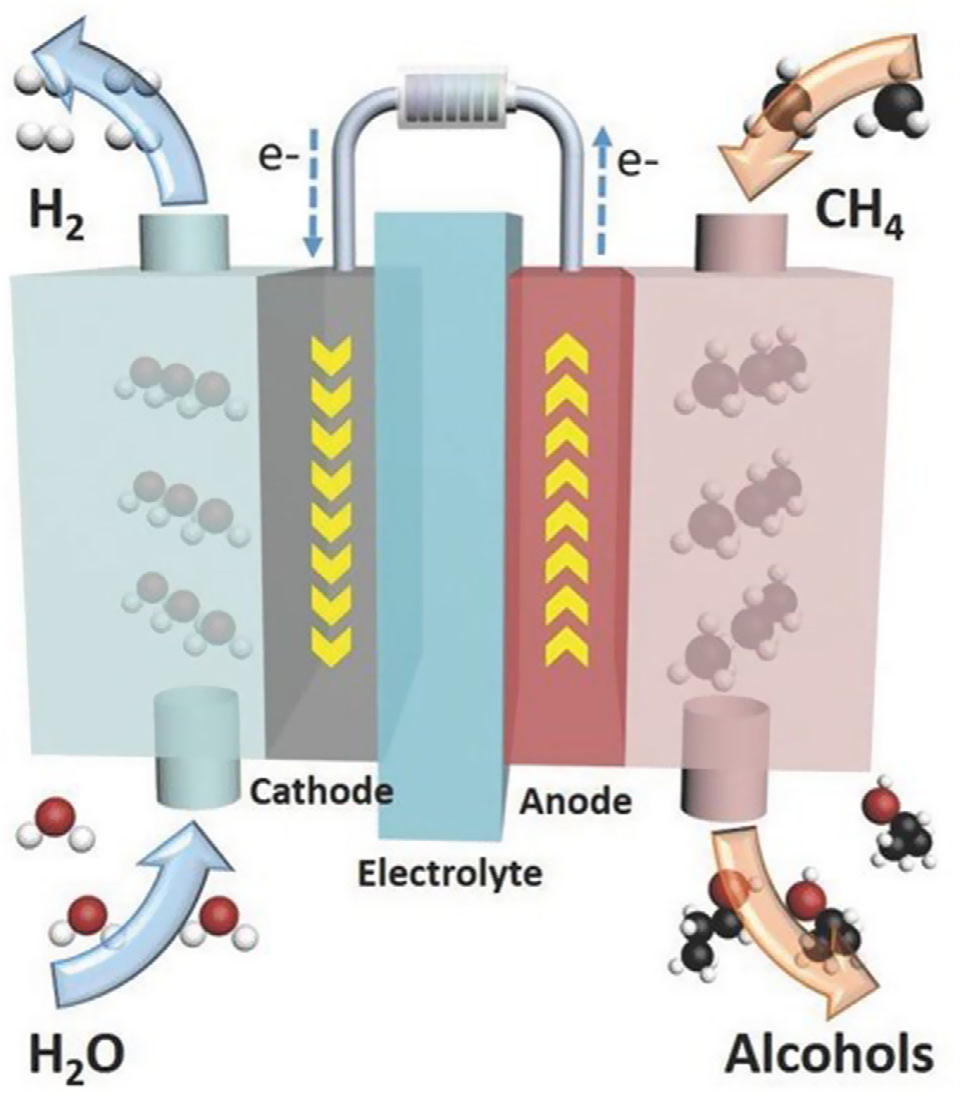
The electrocatalytic oxidation of methane. Reproduced with permission.^[^^ 19 ^^]^ Copyright 2017, WILEY‐VCH.

Similarly, oxide‐based catalysts have shown great promise in methane conversion. The ZrO_2_‐NiCo_2_O_4_ quasisolid solution nanowire catalyst exemplifies this potential by achieving methane oxidation to a suite of valuable products, including propionic acid, acetic acid, and acetone. This system operates without the need for high temperatures or noble metals, achieving a conversion efficiency of 47.5% over 20 h of operation. The success of this catalyst lies in its unique nanostructure, which enhances the availability of active sites and promotes efficient oxygenate production,^[^ ^18^ ^]^ same authors presented similar work on operating such cell (**Figure** **8**) at potential of 2.0 V, and after 12 h of operation, there was almost 40% conversion of methane achieved, where they oxidized CH_4_ to 1‐propanol and 2‐propanol via an electrochemical method.^[^ ^19^ ^]^

Additionally, cobalt oxide (Co_3_O_4_) combined with ZrO_2_ nanotube supports has proven to be another highly effective system. The nanostructured Co_3_O_4_‐ZrO_2_ composite facilitates POM into higher alcohols, with a production rate of 2.42 mmol g^−1^ h^−1^, driven by the synergistic interaction between the Co_3_O_4_ and ZrO_2_ components. The high specific surface area and efficient electron transport in this composite further enhance its catalytic activity.^[^ ^20^ ^]^ Chen et al. reported that focusing on the interplay between catalyst design, reactor configuration, and process efficiency, using Co_3_O_4_‐ZrO_2_ composites further highlights the efficiency of oxide‐based catalysts in producing higher alcohols under ambient conditions.

Building on these catalytic innovations, noble metal systems such as platinum‐based catalysts still demonstrate exceptional capabilities in methane functionalization. Pt^(II)^‐based aqueous systems provide a highly selective route for methane oxidation to methanol, achieving a 70% selectivity for methanol production. The process leverages electrochemical regeneration of Pt^(IV)^ oxidants, eliminating the need for stoichiometric oxidants and enabling sustained activity under mild conditions. This approach highlights the potential for integrating electrocatalysis into practical applications by overcoming the economic and operational barriers associated with traditional catalytic cycles.^[^ ^21^ ^]^ Similar to platinum‐based catalysts, Godoi et al. presented PdCu/C system, for example, which demonstrates high activity for POM in alkaline fuel cells (FCs), producing methanol, dimethyl ether, and formates. Differential mass spectroscopy reveals that the Pd_50_Cu_50_/C combination achieves the highest methane oxidation rates, leveraging the synergy between palladium and copper to improve water activation and C—H bond scission. This system also enables energy co‐generation, offering a dual benefit of chemical production and power generation.^[^ ^22^, ^23^ ^]^

Reactor design plays a critical role in translating these catalytic advances into scalable systems. Solid oxide electrolyzers (SOEs) have emerged as a particularly effective platform for methane electrocatalysis. By utilizing Sr_2_Fe_1·5_Mo_0·5_O_6_‐δ (SFMO) catalysts, SOEs have demonstrated selective oxidative coupling of methane (OCM) to ethylene with a C_2_ selectivity of 81.2%. This system operates at high temperatures suitable for SOE technology but benefits from the ability to regulate methane activation through applied potentials and the intrinsic stability of SFMO catalysts against coke formation. These features make SOEs a promising candidate for industrial‐scale applications,.^[^ ^24^ ^]^ Zhu. et al. presented a similar SFMO‐based system. By incorporating nanoscale metal‐oxide interfaces on porous SFMO scaffolds, researchers achieved a methane conversion rate of 41% at 850 °C. The interface facilitates efficient C—H activation and prevents coking, making it highly stable under extended operation.^[^ ^25^ ^]^ The study by Chen et al. presents a novel solid oxide FC (SOFC) operating on nearly dry methane (≈3.5 vol% H_2_O) at 500 °C, achieving a peak power density of 0.37 W cm^−^^2^. The SOFC design integrates a PrBa_0·5_Sr_0·5_Co_1·5_Fe_0·5_O_5_+δ (PBSCF) nanofiber‐based cathode, a BaZr_0·1_Ce_0·7_Y_0·1_Yb_0·1_O_3_–δ (BZCYYb)‐based multifunctional anode, and a Ce_0·90_Ni_0·05_Ru_0·05_O_2_ (CNR) catalyst layer for reforming methane into H_2_ and CO. This synergistic combination facilitated effective steam reforming and electrochemical oxidation, yielding high selectivity for H_2_ (99%) and CO (97%) at 500 °C. The catalyst demonstrated robust coking resistance, maintaining performance over 550 h of continuous operation without degradation. The work highlights the synergistic role of Ru and Ni cations in activating CH_4_ and H_2_O for efficient methane reforming, showcasing the potential of advanced catalysts for intermediate‐temperature SOFCs.^[^ ^26^ ^]^

In contrast to SFMO catalyst‐based solid oxide electrolysis systems, Santos et al., confirmed that advances in reactor design have been crucial in translating catalytic innovations into practical applications. Solid electrolyte membrane reactors (SEMRs) and alkaline anion exchange membrane FCs (AAEMFCs) have emerged as leading platforms for methane electrocatalysis. In AAEMFCs, catalysts like Pt/C and Pd/C enable POM to convert methanol and formate at room temperature. Pt/C shows a high conversion efficiency of 20% at 0.3 V, while Pd/C achieves 17.5% at 0.15 V, with products being quantified through Raman spectroscopy. These reactors demonstrate the feasibility of co‐producing electricity and valuable chemicals, particularly in systems where methane serves as a fuel.^[^ ^27^ ^]^

Another innovative reactor configuration involves SEMR‐FCs, which combine methane oxidation with electricity generation. These systems achieve methane‐to‐methanol conversion rates exceeding 1.85 mol L^−1^ h^−1^, leveraging Cu‐complex catalysts under open‐circuit conditions. The integration of methane conversion and energy generation in a single device underscores the potential for creating multifunctional systems that maximize resource utilization.^[^ ^28^ ^]^ The breadth of products achievable through methane electrocatalysis is a testament to the versatility of this approach. Ethanol, methanol, acetic acid, and ethylene are just a few examples of value‐added chemicals produced through these processes.

Despite these advancements, challenges remain in optimizing catalyst stability, improving conversion efficiencies, and reducing operational costs. For example, the chemical stability of alkaline earth metal‐based catalysts in carbon‐rich environments and the susceptibility to overoxidation in oxidative coupling reactions highlight areas for further investigation.^[^ ^24^ ^]^ Addressing these challenges through continued innovation in catalyst design, reactor engineering, and system integration will be crucial for advancing methane electrocatalysis as a commercially viable technology.

In short, electrocatalytic methane conversion represents a paradigm shift in the utilization of natural gas, offering a pathway to sustainable and efficient production of value‐added chemicals. The combination of advanced catalysts, innovative reactor designs, and precise reaction control underscores the transformative potential of this technology, paving the way for its integration into future energy and chemical production systems.

The integration of electrified methane conversion technologies with renewable energy sources could be achieved through several practical strategies. These include leveraging the dynamic response capabilities of systems like electrochemical cells to match fluctuating power inputs, incorporating energy storage solutions (e.g., batteries or hydrogen) to buffer variability, and using smart grid technologies to enable demand‐response operation. Additionally, the modular and distributed nature of many electrified systems makes them well‐suited for deployment near renewable energy generation sites, enhancing overall process efficiency and reducing transmission losses. Such approaches can facilitate flexible, low‐emission industrial processes aligned with renewable energy availability.

However, a significant gap remains in the current body of work, as long‐term catalyst stability is rarely addressed. For these technologies to attract industrial interest and support further development, comprehensive studies on durability and operational lifetime are essential.

### 2.2 Future Outlook for Electrocatalytic Methane Conversion

Ionic liquids (ILs), often referred to as molten salts, are emerging as powerful tools in methane conversion and coupling reactions, particularly as electrolytes in electrolysis. Defined as compounds composed entirely of ions, ILs have been a focus of intense research over the past two decades due to their tunable properties, including their ability to remain liquid at temperatures below 100 °C. Since Paul Walden first reported ILs in 1914, they have developed into a major area of study across disciplines such as chemistry, materials science, and environmental science, with over 5,000 articles published in the past two decades, and have recently gained back a huge interest.^[^ ^29^ ^]^ ILs unlocked many new reaction pathways and mechanisms for methane conversion. But ILs can not only work as an effective electrolyte or precursor for catalyst formation, but also as an effective methane‐soluble medium for methane trapping or transporting.

**Figure 9:**
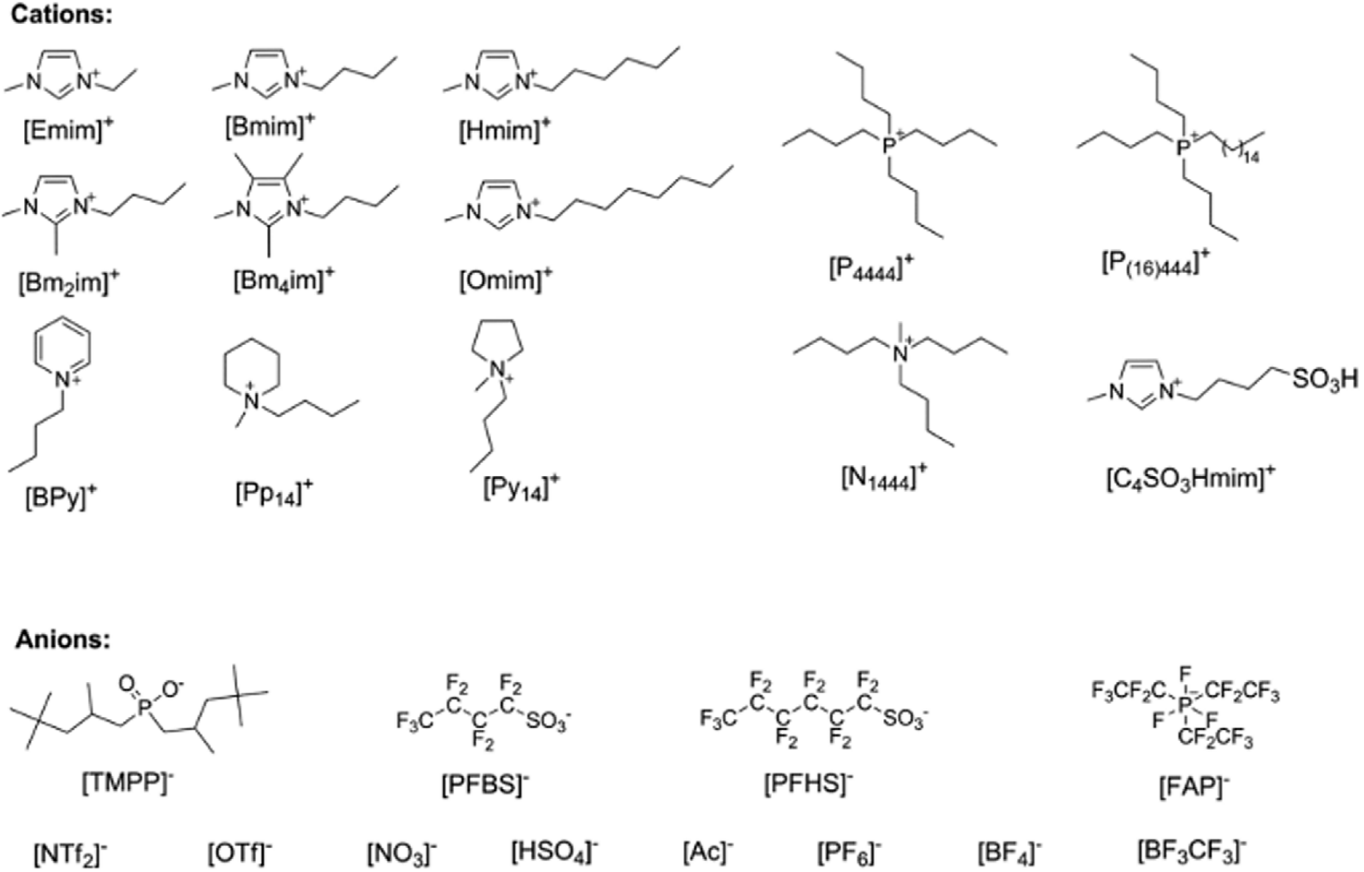
ILs reported for methane dissolution. Reproduced with permission.^[^ ^31^ ^]^ Copyright 2025, American Chemical Society.

Initial studies have highlighted ILs’ versatility in methane conversion (**Figure** **9**). For example, Cheng et al.^[^ ^30^ ^]^ demonstrated a method to prepare highly active ternary systems comprising ILs, inorganic platinum compounds, and sulfuric acid for the direct conversion of methane to methanol. Typically, inorganic Pt compounds such as PtCl_2_ or PtO_2_ are insoluble in organic or aqueous solutions and even in concentrated acids. However, the study found that heating Pt compounds in ILs, followed by dissolution in sulfuric acid, created a homogeneous solution that significantly enhanced catalytic activity. Methanol concentrations of 0.17 M were achieved using PtCl_2_ + [1‐mim][Cl] in 96% H_2_SO_4_, a performance approximately five times higher than with traditional systems like (Bpym)PtCl_2_.

Building on this, Whang et al. demonstrated the ability of ILs to electrochemically activate methane at the IL/Pt electrode interface. In this system, the C—H bond in methane was broken to form a methyl radical through the generation of bis((trifluoromethyl)‐sulfonyl)‐amide ([NTf_2_]) radicals. Using 1‐butyl‐1‐methylpyrrolidinium bis((trifluoromethyl) sulfonyl) amide ([Bmpy][NTf_2_]), methane was oxidized anaerobically at the platinum‐[Bmpy][NTf_2_] interface, forming methyl radicals that are crucial for downstream applications such as organ fluorination.^[^ ^31^ ^]^

More recently, they continued the work on electrocatalytic methane conversion in IL electrolyte, converting it into methanol under mild conditions using an oxygen‐vacancy‐rich V_2_O_5_ (Ov‐V_2_O_5_) anode and an IL electrolyte, 1‐butyl‐3‐methylimidazolium tetrafluoroborate ([BMIM]BF_4_), in a non‐diaphragm electrochemical system.

**Figure 10:**
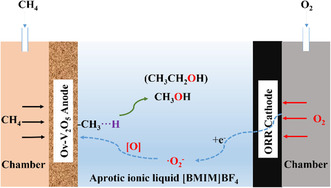
Mechanistic pathway of methane conversion using oxygen‐vacancy‐rich anode in IL electrolyte. Reproduced with permission.^[^ ^32^ ^]^ Copyright 2025, Elsevier.

They have clarified the mechanistic underpinnings of IL‐based electrocatalytic methane conversion (**Figure** **10**), particularly highlighting the role of electrogenerated oxygen species in selective oxidation. In systems using aprotic ILs such as [BMIM]BF_4_, active oxygen species like superoxide (⋅O_2_^−^) and peroxide anions (O_2_^2^^−^) are generated via the oxygen reduction reaction at cathodic potentials of −0.85 and −1.20 V, respectively. These species participate in the oxidation of methane adsorbed on anodic catalysts such as oxygen‐deficient V_2_O_5_ (Ov‐V_2_O_5_), which contains V^4+^ sites that facilitate CH_4_ chemisorption and lower the activation barrier, as supported by density functional theory (DFT) calculations showing an oxygen vacancy formation energy of 2.30 eV. Under optimized conditions, methanol was produced at a rate of 352.5 μmol g_cat^−1^ h^−1^ with a FE of 61.1%. The IL medium enhances the stability of reactive oxygen species by suppressing their protonation and disproportionation, offering better control over reaction selectivity. However, challenges remain in aligning the kinetics of methane activation with the oxygen supply, as excess ⋅O_2_^−^ can be lost to side reactions, reducing overall efficiency. These findings demonstrate both the potential and the complexity of IL‐based systems for carbon‐efficient methane upgrading under mild conditions.^[^ ^32^ ^]^

ILs are paving the way for new reaction pathways and mechanisms in methane conversion. Their tunable properties allow for the engineering of highly specific systems and processes, making them ideal for applications where unique solvent or electrolyte characteristics are desirable. Additionally, their environmentally friendly nature, along with easy recovery and regeneration, positions ILs as a superior alternative to conventional solvent systems. With continued advancements, ILs hold great promise for revolutionizing methane conversion and other electrochemical applications. There are still a few technical barriers for IL‐based methane conversion, which mostly include low methane solubility, limited ionic conductivity, poor long‐term stability of ILs, and catalyst deactivation over time.

### 2.3 Methane Activation in the Electric Field

Recent research has demonstrated significant advancements in electrocatalytic methane conversion using electric fields, emphasizing their role in direct activation and enhancement of reaction efficiencies. Some publications have reported that methane conversion improvement can be achieved by applying a lower external E‐field.^[^ ^33^, ^34^, ^35^ ^]^ For instance, Bai et al. illustrated that microwave‐assisted methane dehydroaromatization with Mo/ZSM‐5 catalysts achieved up to 18% methane conversion at 550 °C under an electric field, compared to over 800 °C required in conventional thermal systems. The non‐thermal effects of microwave irradiation created highly localized “hot spots” on the catalyst surface, effectively aiding the conversion process to yield C2 hydrocarbons and aromatics such as benzene. Authors reported the issue of carbon deposition under microwave‐assisted conditions. However, coke formation still presents a significant challenge due to its impact on catalyst performance and system stability. Interestingly, their observations revealed that the power required to maintain the target temperature dropped significantly to 30–50 W after initiating the reaction. This effect is attributed to coke acting as a strong microwave absorber, thereby influencing the local heating profile. However, since the coke formation rate varied between experiments, only the initial power input before reaction onset was used for comparison. This variability further underscores the need for careful control and potential mitigation strategies to manage carbon deposition during electric field‐assisted methane conversion.^[^ ^36^ ^]^

**Figure 11:**
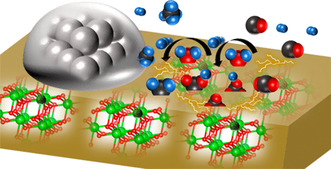
Schematic illustrating the mechanism of dry reforming of CH4 over Ni/La‐ZrO_2_ catalyst in an electric field. (Atoms colors: La atoms ‐> dark green; Zr atoms ‐> light green; Ni atoms ‐> gray; C atoms ‐> black; O atoms ‐> red; and H atoms ‐> blue). Reproduced with permission.^[^ ^37^ ^]^ Copyright 2025, American Chemical Society.

Nakano et al.^[^ ^37^ ^]^ reported that in dry reforming of methane (DRM), applying a direct current electric field to Pt/CeO_2_ catalysts facilitated low‐temperature methane conversion at 473 K. The electric field promoted the interaction between CH_4_ and surface lattice oxygen at the Pt–CeO_2_ interface, increasing CO_2_ activation through carbonate intermediates and enhancing CH_4_ dissociation via surface oxygen vacancies. The application of an electric field significantly reduces the apparent activation energies for CH_4_ and CO_2_ (from 66.1 and 62.3 kJ mol^−1^ to 8.2 and 12.1 kJ mol^−1^, respectively), indicating a fundamentally different, non‐thermal reaction pathway. Central to this mechanism is the promotion of proton conduction on the catalyst surface via the Grotthuss mechanism, wherein protons from adsorbed water and hydroxyl groups “hop” across the surface and collide with methane molecules to form a CH_3_—H—H^+^; transition state, facilitating C—H bond cleavage (**Figure** **11**). Turnover frequency analyzes reveal that catalytic activity correlates more strongly with the Ni perimeter than total surface area, emphasizing the role of the Ni/La‐ZrO_2_ interface as the primary active site. Isotopic labeling experiments with ^18^O_2_ confirm the participation of lattice oxygen in a Mars–van Krevelen redox cycle, while in situ diffuse reflectance infrared Fourier transform spectroscopy data show reversible water rotation bands (850 cm^−1^) exclusive to electric field conditions, corroborating surface proton dynamics. An inverse kinetic isotope effect observed with CD_4_ supports proton‐assisted activation of methane, and DFT calculations show enhanced CO_2_ adsorption and bicarbonate formation at oxygen‐deficient La‐ZrO_2_ sites. Together, these findings demonstrate that the electric field promotes methane and CO_2_ activation through a coupled protonic–redox mechanism at significantly reduced temperatures.

Similarly, Yabe et al.^[^ ^38^ ^]^ demonstrated that Ni/La‐ZrO_2_ catalysts under an electric field achieved DRM with a marked reduction in activation energy. The electric field synergized with surface protonic mechanisms, where proton collisions at the catalyst interface enabled the formation of transition states conducive to CH_4_ conversion, producing synthesis gas with reduced coke deposition.

In another study, Han et al.^[^ ^39^ ^]^ explored OCM using TiO_2_/ZSM‐5 core‐shell particles. The application of an electric field activated oxygen species at the TiO_2_‐zeolite interface, facilitating the production of light olefins such as ethylene and propylene. The selectivity toward desirable hydrocarbons was enhanced, reducing deep oxidation side reactions. An experiment at low reaction temperature of 150 °C, resulted in high methane conversion (18.4%) with selectivity 8.5% toward C_2_H_6_ and 15.7% toward C_2_H_4_, whereas the selectivity toward CO and CO_2_ was at 64.2% and 3.4%, respectively.^[^ ^40^ ^]^ Zhao et al. similarly investigated Ce‐doped TiO_2_ catalysts, where electric fields induced dynamic lattice phase transitions from anatase to rutile, improving CH_4_ oxidation efficiency. This transformation also promoted Pd reduction from Pd^2^^+^ to Pd^0^, an active state for catalytic methane oxidation.

Electrostatic fields have also been shown to directly activate methane through unique mechanisms. Geng et al.^[^ ^41^ ^]^ highlighted a concerted double C—H bond insertion mechanism using Cu^+^‐carbide catalysts in the presence of an electric field, enabling the direct conversion of methane to ethylene. This mechanistically novel approach bypassed the need for intermediate syngas formation, offering an energy‐efficient pathway.

Collectively, these studies underscore the transformative potential of electric fields in electrocatalytic methane conversion, enabling novel reaction pathways, reducing operating temperatures, and enhancing catalytic efficiencies. The resulting products, ranging from syngas to value‐added hydrocarbons such as ethylene, propylene, and aromatics, highlight the versatility and sustainability of electric field‐assisted catalytic systems. These advancements pave the way for cleaner methane utilization technologies, significantly contributing to carbon‐neutral energy and chemical production.

Electric field‐assisted methane conversion faces several critical challenges that must be addressed to advance the technology. A key barrier is the limited molecular‐level understanding of how electric fields influence reaction pathways, activation energies, and transient intermediates, particularly under operando conditions. Most current studies rely on simplified models or ex situ analyses, which fail to capture the dynamic and localized effects of electric fields. Moreover, the lack of standardized experimental setups hinders reproducibility and cross‐comparison between studies. To overcome these challenges, the field requires the development of advanced in situ characterization techniques and multiscale simulations, alongside systematic investigations into the effects of field strength, orientation, and electrode composition. Bridging these knowledge gaps will be essential for optimizing reactor configurations and enhancing the efficiency and selectivity of electric field‐assisted processes.

## 3 Methane Conversion in Plasma

Among many innovative technologies for the conversion of CH_4_ to more energy‐dense fuels, plasma‐assisted methane reforming (PAMR) technology has garnered widespread interest because of its promising benefits associated with gentle reaction conditions. Plasma, a distinctive conductive fluid existing in the form of ionized gas, is composed of ions, electrons with positive and negative charges, atoms and molecules in various neutral, excited, or ground states, free radicals, and photons. A defining characteristic of plasma is its electrical neutrality, maintained whether partially or entirely ionized. Temperature is the parameter which categorizes plasma into two main types: high temperature plasma and low temperature plasma, which also divides into thermal and non‐thermal plasma (NTP). Plasma technology exhibits compelling attributes toward methane reforming, such as achieving higher conversion rates and product selectivity while operating at lower temperatures, leading to a notable reduction in carbon deposition throughout the process. Furthermore, the temperature and energy densities within specific plasma phases far surpass those attainable in conventional catalytic processes, reaching remarkable levels of intensity. These attributes refer to NTP that has highly energetic electrons at comparatively lower overall temperatures compared to the thermal plasma.^[^ ^42^, ^43^ ^]^ Most represented plasma types for methane conversion are presented in **Table** **2**, and an extensive comparison of representational recent works is presented in **Table** **3**.

**Table 2:** Most represented plasma types for methane conversion.

Plasma Type	Temperature	Mode of Operation
Spark Plasma	High (localized)	Triggered by high voltage
Pulsed Plasma	Moderate to High	Short pulses of energy
DBD Plasma	Low (non‐thermal)	Alternating current, dielectric barrier

**Table 3:** Comparison of different plasma‐assisted methane reforming methods.

Reference	Type of Plasma Used	Reaction Conditions	SEI (kJ/mol CH_4_)	Products from CH_4_
Sun et al.^[^ ^49^ ^]^	NPD	50 mL min^−1^ CH_4_, 1 kHz, 100–350 ns, 16–20 kV	245–702	C_2_H_2_, C_2_H_4_, C_2_H_6_, C_3_H_8_, H_2_, soot
Delikonstantis et al.^[^ ^47^ ^]^	NPD	CH_4_:H_2_ (1:1), 11 W, 1–100 kHz, pressure varied	870	Ethylene, acetylene, H_2_, C
Scapinello et al.^[^ ^46^ ^]^	NPD	CH_4_:D_2_ or CD_4_:H_2_ at 1–5 bar, 20–24 W, 3 kHz	245–702	Ethylene, ethane, acetylene, H_2_, D_2_
Huang et al.^[^ ^48^ ^]^	Nanosecond pulsed plasma	1% CH_4_ in Ar, 100 mL min^−1^, 17 Torr, 1 kHz	44.352 (Low methane %)	C2H2, C2H4, C2H6
Wang et al.^[^ ^45^ ^]^	Nanosecond pulsed DBD	CH_4_:CO_2_ (1:1), 50 sccm, 55.7 W, 10 kHz	247–315	H_2_, CO (syngas), small C_2_‐C_4_ hydrocarbons
Nishimura et al.^[^ ^53^ ^]^	DBD (non‐thermal)	CH_4_/CO_2_/Ar feed, 3.6 kV, 373 K, 77 mL min^−1^	–	Acetic acid, formic acid, C_2_‐C_4_ acids
Liu et al.^[^ ^54^ ^]^	DBD plasma with catalyst	CH_4_: He or CH_4_ only, 54 W, atmospheric pressure	–	Ethylene, ethane, acetylene, H_2_
Miao et al.^[^ ^55^ ^]^	DC glow discharge	CH_4_/O_2_/N_2_ & CH_4_/CO_2_/N_2_, 100 mL min^−1^, ambient pressure	67.200 (Low methane %)	Ethylene, C_2_^+^ hydrocarbons, H_2_, CO
Jurković et al.^[^ ^44^ ^]^	Spark plasma	CH_4_/CO_2_, 100–200 cm^3^ min^−1^, 90–150 V	up to 367	H_2_, CO, small acetylene content, some coke

### 3.1 Spark Plasma

Recent advancements in thermal‐catalytic DRM have highlighted the recurring issue of catalyst deactivation due to coking at high reaction temperatures, which limits efficiency. Plasma‐based DRM offers a solution, as it operates at lower temperatures, reducing coking and enhancing stability. While most studies focus on plasma‐only systems, some incorporate catalytic materials around the plasma channel to leverage the heat generated in the plasma for thermo‐catalytic reactions.

In one study by Lašič et al.,^[^ ^44^ ^]^ an atmospheric pressure spark discharge plasma reactor was designed to introduce gases through hollow electrodes into a plasma channel, dispersing them outward through a catalyst made of porous alumina foam wash‐coated with 15 wt% Ni/Al_2_O_3_. Plasma‐only experiments revealed that higher CH_4_ content in the feed increases H_2_ content in syngas but also raises coke formation and reduces CH_4_ conversion while improving CO_2_ conversion. However, catalytic tests showed lower conversions than plasma‐only experiments due to the reverse reactions facilitated by plasma‐generated heat, which often falls short of achieving the thermal equilibrium needed for higher conversions. Effective insulation and elevated catalyst temperatures could mitigate this issue and enhance performance. Notable findings include CH_4_ and CO_2_ conversion rates of 85% and 75%, respectively, with syngas as the predominant product.

### 3.2 Pulsed Plasma

Recent advancements in pulsed discharge plasma technology have demonstrated its potential for efficient non‐oxidative methane (CH_4_) conversion to hydrogen (H_2_). In a needle‐to‐plate discharge reactor, experiments with nanosecond and microsecond pulsed spark discharges revealed significant differences in conversion and product yields. Using a nanosecond pulsed discharge (NPD) with a 10 mm gap length, CH_4_ conversion reached 54.5% with an H_2_ yield of 17.9%. In contrast, the microsecond pulsed discharge achieved a much higher CH_4_ conversion of 91.2% and an H_2_ yield of 38.4% at a 6 mm gap length. Increased gas flow rates reduced CH_4_ conversion and H_2_ yield due to shorter residence times in the plasma, limiting electron‐molecule collisions. The findings highlight the superior efficiency of microsecond pulsed discharges for methane‐to‐hydrogen conversion, with results dependent on discharge gap lengths and gas flow rates.^[^ ^45^ ^]^

The study by Scapinello et al.^[^ ^46^ ^]^ focuses on the mechanism of non‐oxidative methane coupling in NPD reactors, highlighting its efficiency in methane activation and product selectivity. Their research demonstrates how elevated pressures and co‐feeding hydrogen improve methane conversion rates and ethylene yields, with selectivity reaching around 20% per pass under optimized conditions. The study emphasizes the dual role of plasma in initiating methane dissociation and controlling intermediate reactions to favor ethylene over acetylene. A key advantage of this work lies in its use of isotope tracers to elucidate reaction pathways, providing a deeper understanding of radical chemistry and selective product formation.

Delikonstantis et al.^[^ ^47^ ^]^ investigate a plate‐to‐plate NPD reactor configuration, emphasizing its suitability for non‐oxidative methane coupling. Their research demonstrates how proper load‐impedance matching enhances energy utilization and product yields. Despite slightly lower olefin yields compared to coaxial setups, the plate‐to‐plate reactor offers significant energy savings and improved operational stability, reducing carbon deposition and extending operating life. Methane conversion rates of up to 34% are reported, with ethylene and acetylene as dominant products. This work highlights the potential of reactor design optimization in achieving high‐performance methane reforming at reduced energy costs.

Huang et al.^[^ ^48^ ^]^ present an innovative energy pooling mechanism for catalyst‐free methane activation in an argon‐methane mixture using NPD. Their experiments reveal how argon ions and metastable species store energy during the pulse‐on period, sustaining the formation of reactive intermediates during the pulse‐off phase. This mechanism increases the methane dissociation degree by an order of magnitude compared to direct electron impact processes, achieving notable selectivity for hydrogen and light hydrocarbons such as ethylene and acetylene. The main advantage of this study lies in its use of real time, in situ diagnostics and kinetic modeling, providing a clear mechanistic insight into plasma–methane interactions at low temperatures.

Sun et al.^[^ ^49^ ^]^ explore methane conversion across diffuse, filamentary, and spark discharge regimes in NPD reactors, each offering unique product distributions. Their findings indicate that filamentary discharges favor the production of ethane and ethylene, while spark discharges lead to higher acetylene yields due to the dominance of thermal chemistry. Methane conversion rates range from 2.2% in low‐energy diffuse regimes to over 74% in spark discharges. This work underscores the importance of discharge regime control in tailoring product selectivity and highlights the potential of high‐energy regimes for industrial applications despite increased soot formation. The study's advantage lies in its comprehensive analysis of discharge dynamics and optical emission spectroscopy, linking plasma properties to methane conversion outcomes.

### 3.3 Dielectric Barrier Discharge (DBD)

Plasma can be categorized into low and high‐temperature states, depending on the internal electron temperature. In the domain of low‐temperature plasma, thermal and NTP are differentiated based on thermodynamic equilibrium. One notable demonstration of NTP is the DBD.^[^ ^50^ ^]^

DBDs refer to self‐sustaining electrical discharges occurring within electrode setups featuring insulating material within the discharge path. The dielectric or insulating material is accountable for the induction of NTP at regular pressure. The intention behind introducing an insulating medium into the discharge gap is to restrict current flow and deter the occurrence of sparks and arcs. Most commonly used dielectric materials appear to be quartz, ceramics, glass, Teflon, and silicon rubber.^[^ ^51^ ^]^ DBD reactors are preferred over spark and gliding arc discharges, where maintaining discharge stability becomes challenging and the gases spend limited time in the plasma zone, making it difficult to enforce plasma‐catalyst coupling.

Shivapuji et al.^[^ ^52^ ^]^ investigated the (DBD) plasma as a method for in situ production of Hythane, a methane‐hydrogen blend, which exhibits superior combustion properties compared to pure methane. By optimizing the methane residence time within the plasma zone, the study achieved Hythane compositions with hydrogen fractions of up to 15%. The approach demonstrated enhanced flame speeds, significantly improving thermodynamic efficiency for applications like spark‐ignited engines. The main advantage highlighted in this work is the plasma's ability to create charged intermediate species, contributing to improved reaction kinetics and combustion performance.

Nishimura et al.^[^ ^53^ ^]^ focused on the production of acetic acid from methane and carbon dioxide using a DBD plasma reactor. The study revealed that under NTP conditions, methane activation via electron collisions led to selective production of methyl and other reactive intermediates. However, CO_2_ incorporation into acetic acid was less efficient, indicating the need for improved CO_2_ activation mechanisms. The work emphasized the reactor's potential for carbon fixation and its capability to convert GHGs into value‐added chemicals, presenting an environmentally beneficial pathway.

Liu et al.^[^ ^54^ ^]^ demonstrated the synergistic effects of combining single‐atom Pt catalysts with DBD plasma for nonoxidative methane coupling. The system achieved methane conversion of 39% with a C2 hydrocarbon selectivity of 54% at low temperatures and atmospheric pressure. The study underlined the advantages of single‐atom catalysts in enhancing the selectivity and reducing coke formation compared to traditional nanoparticle‐based catalysts. This integration of plasma and catalysis presents a promising route for efficient methane valorization under mild conditions.

Miao et al.^[^ ^55^ ^]^ explored methane coupling in microstructured DBD reactors to produce ethylene and longer‐chain hydrocarbons. Their system achieved methane conversions of up to 75% with carbon selectivity toward C2+ products as high as 90%, alongside energy efficiencies exceeding 80%. The innovative microreactor design allowed for low‐energy discharge at atmospheric pressure and ambient temperature, significantly reducing operational costs and energy losses. This work highlights the feasibility of scalable and energy‐efficient methane conversion technologies for industrial applications.

These studies collectively illustrate the versatility and advantages of DBD plasma in methane conversion, spanning applications from sustainable fuel production to carbon fixation and high‐value hydrocarbon synthesis.

## 4 Electro‐Thermal Methane Conversion

The most prevalent methane conversion method toward syngas is SMR, which is a highly endothermic process. Despite the fact that modern SMR units, when integrated into the large industrial plants with extensive heat recovery, are capable of operating at near 95% energy efficiency,^[^ ^56^ ^]^ standalone fired reformers typically operate at a considerably lower efficiency of near 50%. Alternative methods for the production of syngas (or hydrogen) from methane include other endothermic processes, such as dry reforming, cracking, etc., with the efficiency of these processes being limited by the constant supply of heat to the catalytic sites within the reactors.

The electrification of thermal methane conversion entails the substitution of conventional heat sources with electric‐based technologies. This transition not only facilitates the incorporation of renewable energy into methane processing but also has the potential to overcome limitations of combustion and brings the number of advantages in terms of enhanced energy efficiency, more accurate process control, reduced carbon intensity, and minimized environmental impact.

### 4.1 Joule‐Heating

Nevertheless, the relationship between electrical current and generated heat has been known for nearly two centuries. Joule heating, also known as resistive heating or Ohmic heating, is considered an innovative electrification technique for thermal methane conversion. Apart from electrical heating elements in chemical reactors, which can operate both externally and internally, the most innovative application of resistive heating is direct Joule heating of the internal surface of narrow tubular reactors or bed particles in both fixed‐ and fluidized bed reactors.^[^ ^57^ ^]^ Direct Joule heating operates with exceptionally high energy efficiency, reaching 97–100%.^[^ ^58^ ^]^ This enables the attainment of high heating rates (up to 1000 °C h^−1^) and high operational temperatures (up to 1200 °C).^[^ ^59^ ^]^

Wismann et al.^[^ ^60^ ^]^ demonstrated the prototype, supported by a computational fluid dynamics model and characteristic timescale analysis, which illustrated how direct Joule heating of the tubular reactor used for SMR can overcome limiting thermal conductivity across the catalyst, thereby rendering heat transfer the least limiting mechanism, while reactor performance is governed by diffusion. The experimental setup was constructed using a 6 mm conductive Fe–Cr–Al tube, which was coated on the internal surface with a 130 μm‐thick zirconia‐based wash coat. The wash coat was impregnated with a nickel nitrate solution, reduced, passivated, and reduced once more in situ prior to testing. It was demonstrated that the kinetic, mass, and heat‐transport interplay mechanisms are radically changed in the electrified SMR reactor, leading to considerably higher efficiency than that of conventional SMR reactors.

Another interesting application of direct Joule heating lies in the electrification of fluidized bed reactors.^[^ ^57^ ^]^ When the fluidized bed consists of electrically conductive particles, which allow current to flow between the electrodes, the particles are being heated due to their electrical resistance. Such heating pattern allows high heat transfer rate while keeping the bed temperature uniform, maintaining very high energy efficiency. Fluidized bed reactors, heated by direct Joule heating, known also as electrothermal fluidized bed (ETFB) reactors, ETFB are widely accepted in industry mostly for graphite and coke processing. With their advantages, ETFB reactors are also applied for the energy‐extensive methane pyrolysis process.^[^ ^61^ ^]^

The potential expansion of ETFB reactor applications to other fields is currently under investigation, with a particular focus on overcoming the inherent challenges posed by the necessity of the bed particles to be simultaneously chemically inert and electrically conductive. Recently completed Industrial Chair project “EcoGas” sponsored by TotalEnergies and the Region of Normandy, which was dedicated to “Electrified Conversion of biogas to clean energy vectors: H_2_, E‐Fuel & biofuel components”,^[^ ^61^ ^]^ resulted in the development and construction of an ETFB reactor for effective biogas conversion. The ETFB reactor for acid gas reduction will be demonstrated on a pilot scale as part of the EU‐funded e‐CODUCT project, which is currently underway.^[^ ^62^, ^63^ ^]^

The lack of precise mathematical models of ETFB represents a significant obstacle to a deeper understanding of heat generation in bed particles. This is due to the inherently challenging nature of measuring local resistance between particles and between particles and electrodes. Furthermore, the effective bed resistance is a function of numerous variables, including particle size and shape, bubble formation, superficial gas velocity, and others, making the consideration of all these very challenging.

### 4.2 Induction and Magnetic Heating

One of the first reports on induction heating for heterogeneous catalysis was published already in 1975 by Burton H. Bartley at Texaco as a proof of concept. To show the capability of electrical induction to heat up the catalyst, he prepared an iron catalyst with silica‐alumina support. Induction was also applied to physically mixed iron and silica–alumina. The temperature dependence of propylene hydrogenation, which occurs only on Fe particles, was used to determine the temperature of Fe particles, and cyclopropane isomerization to propylene, which occurs only on silica–alumina was used to determine the temperature of the silica–alumina. Using the temperature dependence of the conversion of both reactants in the presence of hydrogen, it was possible to show the temperature difference between the iron and silica–alumina. In the case of physical mixture, the temperature difference was 94 °C where the temperature of the iron was 245 °C. The temperature difference was smaller in the case of Fe supported on silica–alumina where it was 58 °C, with the temperature of iron of 280 °C. This demonstration showed that differential heating of dual‐function catalyst is possible by electromagnetic induction applied to ferromagnetic material deposited on the non‐conductive and non‐ferromagnetic support.^[^ ^64^ ^]^ In 1982 induction heating of single catalyst pellet with dispersed nickel phase for reforming reactions was studied at 20 MHz of radio frequency and qualitatively described mathematically using Maxwell's equations. It was concluded that with small Ni particles the predominant mechanism of heating are magnetic and dielectric losses where the latter are increasing with temperature. Joule heating due to induced eddy currents is expected only with Ni crystals larger than several hundred ångströms.^[^ ^65^ ^]^ In fact, in different susceptors different heating mechanisms are present. Induced eddy currents occur in electrically conductive materials which can be ferromagnetic or non‐ferromagnetic. In small enough electrically conductive particles (as is the case on many catalysts) eddy currents large enough to heat up the material cannot be induced. However, in magnetic and superparamagnetic nanoparticles hysteresis losses are large enough to generate heat. Additionally, in superparamagnetic particles another contribution to the generation of heat is Brownian and Néel relaxation mechanisms. Hysteresis losses are only present in magnetic materials when a material is heated above its Curie temperature, it stops being magnetic, and heat generation stops.^[^ ^66^ ^]^ Catalysts used in steam and dry reforming of methane and partial oxidation usually contain nanoparticles of active metal on some kind of support. Therefore, heat generation in these catalysts is not a consequence of eddy currents. On the contrary, when induction is used for graphene or carbon nanotubes (CNT) growth metal sheet is heating up due to eddy currents. Developments in different reactions for methane valorization, where effects of induction were used for heating ale presented next. The main advantages of magnetically induced catalysis are localized heating, providing a large temperature difference between the catalyst and the surrounding medium, high energy efficiency, and the ability to rapidly heat up and cool down, which makes it suitable to use with renewable sources of electricity with changing availability.

In 21st century, induction heating started being investigated also for the valorization of methane. Firstly, for growth of CNT and graphene.^[^ ^67^, ^68^ ^]^ And later for endothermic reactions that can be carried out on a larger scale, such as SMR,^[^ ^69^, ^70^, ^71^ ^]^ DRM,^[^ ^72^, ^73^, ^74^ ^]^ methane decomposition,^[^ ^75^ ^]^ and POM.^[^ ^76^, ^77^ ^]^ Graphene and CNT can be grown by catalytic chemical vapor deposition (CCVD) method where the plate or crucible in which the catalyst is placed is heated by induction while in the case of the other mentioned reactions the catalyst itself is usually heated by induction. For example, Biris et al.^[^ ^68^ ^]^ used the CCVD method to heat up Fe‐Mo/MgO catalyst for growth of double walled CNT where methane was used as carbon precursor, and catalyst was placed in graphite receptacle in the quartz tube. The wall of a quartz tube was cooled with water, and the graphite receptacle was heated by an induction coil with 5 kW and 1.9 MHz. Excellent purity of double‐walled CNT of 95% was achieved after purification. Graphene can be grown by decomposition of methane on copper foil that is heated by induction to 1035 °C, as shown by several reports.^[^ ^67^, ^78^, ^79^ ^]^

#### 4.2.1 Steam Methane Reforming

**Figure 12:**
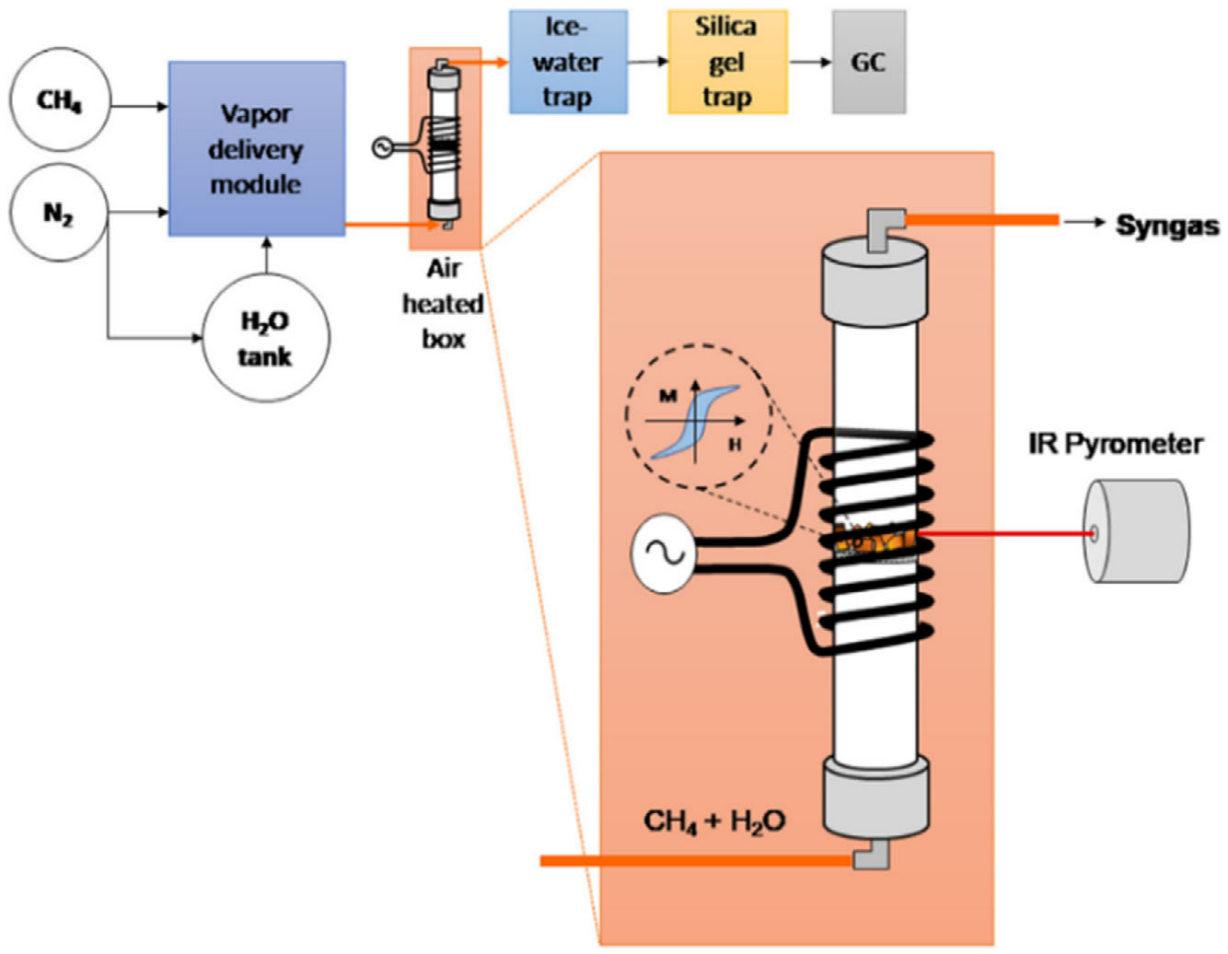
Experimental setup for induction heated SMR. Reproduced with permission.^[^ ^71^ ^]^ Copyright 2025, Elsevier.

More extensive research was carried out on induction heating in SMR where high temperatures, between 800 and 900 °C, are needed. Because of high temperature needed for reaction materials with a high enough Curie temperature should be used to ensure efficient heat generation by hysteresis losses since eddy currents in metal nanoparticles on catalysts are not large enough to heat up the catalyst. Example of a setup used for SMR in induction heated reactor is shown in **Figure** **12**. IR pyrometer is used for measuring the temperature because thermocouples or resistance thermometers are usually not suitable to use in alternating magnetic field due to heat generation in them.

Danish researchers (Mortensens et al.) published several works over the years on induction heating applied to SMR over NiCo containing catalysts with different supports.^[^ ^69^, ^70^, ^80^, ^81^ ^]^ In a publication from 2017, they investigated magnetic properties of catalysts with different content of Co and Ni supported on MgAl_2_O_4_.^[^ ^70^ ^]^ Nickel is responsible for methane conversion, and cobalt ensures that the Curie temperature is above 800 °C. This way the reaction can proceed to high conversions of methane. Another function of the alloy is that it prevents oxidation of cobalt phase and preserves the magnetic properties of cobalt. Higher content of cobalt results in a higher Curie temperature. It was shown that NiCo/MgAl_2_O_4_ with 12.6 wt% of Ni and 9.0 wt% of Co has the best balance between magnetic properties and catalytic activity. In the power range of 1200 to 1600 W (at 68 kHz) conversion of methane above 95% was achieved with flow rates of 20 and 30 NL h^−1^ and loading of the catalyst between 40 and 80 mg diluted with MgAl_2_O_4_ support particles. At the highest power and the lowest flow rate, equilibrium conversion was achieved. According to the composition of the reactor outlet at equilibrium the temperature on the surface of the catalyst was at least 780 °C. The main advantage of induction heating is elimination of heat transfer limitations because heat generation occurs directly at catalyst active sites while on industrial scale reactors are heated with burning of natural gas, around 50% of the energy is transferred to the reactor and the rest is recuperated from the flue gas.^[^ ^82^ ^]^

In another work the catalysts were prepared by precipitation method where resulting spinel (M_y_Co_(1‐*x*‐1/2*y*)_Ni_(*x*‐1/2*y*)_Al_2_O_4_ where M = Cu) was converted to metal nanoparticles on alumina support upon calcination and reduction. Two catalysts were extensively characterized and evaluated for induction heated SMR. The first one was Co_0.5_Ni_0.5_/Al_2_O_3_ (17.7 wt% Co and 17.3 wt% Ni) and the second Cu_0.36_Co_0.5_Ni_0.5_/Al_2_O_3_ (0.4 wt% Cu). Curie temperature of materials was determined to be 892 and 875 °C for catalyst without and with copper, respectively. Comparison of methane conversion at the same applied magnetic field strength showed that the catalyst with Cu is 15% more effective than the catalyst without Cu but magnetic properties were very similar. The explanation that was provided is that addition of copper to nickel catalyst promotes activity for SMR. Magnetic field amplitudes in range of 20 to 42 mT were used for catalytic activity evaluation and the frequency of alternating electromagnetic field was always 69 kHz. Long term stability of NiCo catalyst was evaluated by using the catalyst for 300 h and no loss in activity was observed. Conversion of methane was around 90% which corresponds to equilibrium at 715 °C. After characterization of the used catalyst no changes in material were observed compared to the fresh catalyst.

Further, the effect of frequency, coil geometry (height and radius), and insulation type on the catalyst efficiency was studied.^[^ ^81^ ^]^ Again, the catalyst was CoNi on alumina support. Although samarium was added during synthesis in an attempt to improve hysteresis heating of CoNi it was found that in the prepared catalyst Sm was present only on the support and did not contribute to heating of the active phase. The catalyst was denoted as CoNi/Sm_2_O_3_‐Al_2_O_3_ containing 15.6 wt% Co and 15.5 wt% Ni. The content of Sm was 8.15 wt%, but it did not contribute to catalyst activity or magnetic properties or heating of the catalyst. The Curie temperature was determined to be 877 °C. With the same coil geometry and insulation around the reactor, increasing the frequency from 68 to 189 kHz decreased the power needed to reach 90% conversion of methane. At a lower frequency, the power needed was more than 800 W, but at a higher frequency, the required power was around 500 W. Therefore, increasing the frequency resulted in 18% higher efficiency (power for reaction compared to the entire power input). The benefit of increasing the frequency is limited by resistive losses, which become significant as the frequency is increased. When a coil with a smaller diameter (25 mm) was used, the efficiency increased from 16% (with coil with 40 mm) to 23%. With a smaller coil diameter coil tube length is also reduced, which results in smaller power loss due to electrical resistance of the coil. Increasing the length of the coil from 60 to 135 mm with the coil diameter of 25 mm did not have a significant influence on the efficiency. Also changing the insulation from one kind to another did not significantly affect the efficiency of the power delivery to the catalyst.

Mortensen et al.^[^ ^80^ ^]^ demonstrated that adjusting the Co/Ni ratio in metal nanoparticles can tune the Curie temperature, allowing control over the catalyst's maximum operational temperature. They prepared five catalysts with Co/Ni ratios from 0.25 to 2.3, all containing ≈33 wt% metal and nanoparticle sizes of 25–30 nm on Al_2_O_3_ support. In SMR with an applied alternating magnetic field with 195 kHz, the catalysts exhibited varying efficiencies. The catalyst with a Co/Ni ratio of 1/4 (Curie temperature: 617 °C) achieved methane conversion of 53–58% as the field amplitude increased from 15 to 30 mT. In contrast, the catalyst with a Co/Ni ratio of 2/3 (Curie temperature: 795 °C) reached over 90% conversion at 20 mT, as it achieved higher temperatures. The outlet gas temperature was 591 °C for the lower cobalt catalyst and 728 °C for the higher cobalt catalyst. Further increasing the cobalt content required larger field amplitudes due to lower hysteresis area (magnetic hardness of Co) despite higher Curie temperatures. Co‐rich catalysts were also less active in SMR due to reduced nickel content. The hysteresis area increased with cobalt content up to 50% but decreased thereafter, while Curie temperature rose steadily with cobalt.

**Figure 13:**
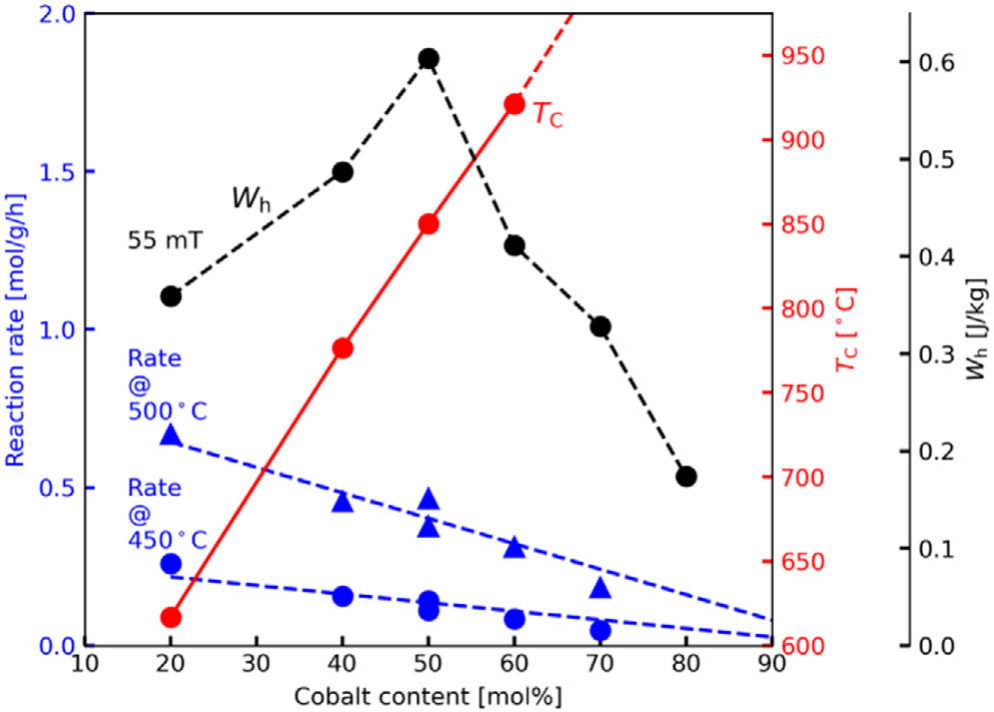
Activity of catalysts with different Co content and their Curie temperature and magnetic hysteresis areas. Reproduced with permission.^[^ ^80^ ^]^ Copyright 2025, American Chemical Society.

These results are summarized in **Figure** **13**. Optimizing Co content enables self‐regulation of reactor temperature via Curie temperature. A concept is more advantageous for exothermic reactions since it can prevent occurrence of hot spots and temperature excursions.

A different preparation method was used by Polleto Dotsenko et al.^[^ ^83^ ^]^ for the preparation of NiCo containing catalysts for induction heated SMR. Conventional impregnation was employed to prepare 3 different catalysts supported on γ‐Al2O3 (1/8” cylindrical pellets). Two catalysts had the same total metal loading (30 wt%) and different Co/Ni ratios, namely 2/3 and 1. The third catalyst had 35 wt% metal loading and Ni/Co ratio of 2/3. The radio frequency of induction was 242 kHz and the maximum power that could be supplied was 2 kW. Specific absorption rate (rate of absorption of energy) of the catalyst was estimated by measuring the temperature at different magnetic field amplitudes. It was found that the absorption rate is higher for the catalyst with Co/Ni ratio of 1. Also, this catalyst performed the best among all 3 catalysts that were used for SMR at gas hourly space velocity (GHSV) of 3000 h^−1^ and applied magnetic field amplitude of 28.5 mT. All 3 catalysts reached equilibrium conversion of methane and the conversion was only limited by the temperature that was reached at the catalyst and not by kinetic limitations.

Besides heating up the catalyst itself, induction can obviously also be used for heating up the reactor. Ma et al.^[^ ^84^ ^]^ constructed a reactor where the energy for heating was delivered to the heat pipe surrounding the packed‐bed reactor by induction. The heat pipe was filled with sodium and was evaporating in the zone where the coil was placed. Condensation of sodium in the upper part of the heat pipe ensured a uniform temperature profile along the heat pipe and reactor. Commercial Ni/Al_2_O_3_ catalyst was used for SMR. The reactor operated at 620 °C and was able to heat up in 250 s.

#### 4.2.2 Dry Reforming

Similar to the works done on SMR, Varsano et al.^[^ ^72^ ^]^ investigated the DRM powered by induction heating. The energy was delivered directly to a Ni_60_Co_40_ alloy, which served as both the catalyst and the heat generator, enabling the heat for the reaction to be generated within the catalytic bed itself. This design eliminates inefficiencies associated with external heat transfer. Using Ni_60_Co_40_ pellets in a continuous‐flow fixed‐bed reactor, temperatures exceeding 850 °C were achieved, facilitating methane conversion and hydrogen production with yields comparable to conventional heating methods. Notably, methane conversions exceeding 70% and yields of CO and H_2_ near 70% were attained at 850 °C.

In 2015, Perez‐Camacho et al.^[^ ^85^ ^]^ applied induction heating of the catalyst for dry and SMR. A stainless steel reactor with 0.1 g of mixed oxide catalyst (Na_0.5_La_0.5_Ni_0.3_Al_0.7_O_2.5_) was used. GHSV was 30 L/(g_cat_ h). Induction heating was provided by a system capable of 2 kW of output power. High conversion of methane (around 90%) was achieved by dry reforming at the highest electrical current applied (around 110 A). Temperature of the reactor surface reached 552 °C (measured by IR probe) at that current and temperature in the catalyst bed was measured by a K‐type thermocouple which showed 860 °C. It was shown that, because of fast response in heating up and cooling down of the reactor, the system is suitable for use with electrical energy from renewable sources such as wind power, where the availability of electricity is changing with time.

Li et al.^[^ ^74^ ^]^ reported the magnetic field‐assisted CO_2_ reforming of methane over a novel hierarchical Co/MgO catalyst in a fluidized bed reactor. The Co/MgO catalyst demonstrated superior CO_2_ and CH_4_ conversions, higher turnover rates, and enhanced resistance to coke deposition compared to a conventional fluidized bed reactor. At 800 °C CO_2_ and CH_4_ conversions were improved by 22% and 30%, respectively, in the magnetic‐assisted fluidized bed (MFB) reactor. The MFB reactor also exhibited a slightly lower apparent activation energy (78.1 vs. 80.4 kJ mol^−1^) and prevented Co nanoparticle transformation into carbon tubes, highlighting the benefits of magnetic field integration for enhanced catalytic performance.

Most recently, bi‐reforming (dry and SMR) of methane was carried out over Cu‐Co and Cu‐Ni‐Co, Cu‐Ni and Cu catalysts heated by induction.^[^ ^73^ ^]^ The catalysts were prepared by the co‐precipitation method and were evaluated for methane reforming reactions in magnetic fields up to 42 mT and a frequency of 69 kHz. By changing the applied field, the temperature of the reaction was controlled. The experiments were performed in temperature range of 400 to 700 °C. The weight hourly space velocity was 5.0 L (g_cat_*h)^−1^ and it was the same for all the experiments. At 700 °C conversion of CO_2_ was 61% and conversion of methane was 92% over Cu‐Co catalyst. Selectivity for CO was almost 60%. Curie temperature of this material was determined to be 390 °C. Higher activity compared to other catalysts was attributed to greater hysteresis area of the Cu‐Co catalyst and larger effect of hysteresis heating although all the catalysts were evaluated at the same temperature range. Temperature of the surface of the reactor was monitored by pyrometer and the middle of the catalyst bed might be heated up to higher temperature which might also contribute to difference in activity with different catalysts. Also, copper catalyst which does not poses magnetic properties was heated up to 700 °C by induced eddy currents. The difference in activity might also stem from different kinetics on different materials.

#### 4.2.3 Partial Oxidation

Patel et al. studied non‐catalytic POM in indirectly induction heated flow reactor.^[^ ^76^, ^77^ ^]^ They designed an induction furnace flow reactor from SiC tube which was placed inside a copper coil. At the middle of the coil a graphite susceptor cylinder was placed and the SiC reactor tube was going through it. Only 500 K could be achieved if no graphite susceptor was used and only the SiC reactor was heated with induction. When a graphite susceptor was used, mechanical contact between graphite disc and SiC reactor, and high thermal conductivity of SiC ensured heat transfer to the interior of the reactor and thus providing the heat for the reaction. The frequency of induction was 0.7 MHz, and the maximum power of the radio frequency generator was 6 kW. Reactor had an internal diameter of 6 mm. The height of the graphite susceptor was 5 mm but hot zone was longer because of high thermal conductivity of SiC. In this setup POM was carried out in temperature range from 1485 to 1592 °C. The influence of temperature CH_4_/O_2_ ratio and flow rate was investigated. It was found that at flow rate (3000 sccm) reactor was blocked with coke in 3 min while at 9000 sccm, it was possible to run the reaction for 60 min. Complete conversion of O_2_ and high conversion of methane (over 80%) was achieved. The main advantage of the reactor was the ability to rapidly change the temperature in the reactor in response to changing flow conditions or other conditions in the reactor, which can occur due to carbon deposits.^[^ ^77^ ^]^

## 5 Conclusion

### 5.1 Energy Outlook and Scalability

**Electrocatalytic conversion**, while promising for its selectivity and operation under ambient conditions, currently suffers from low overall energy efficiency (<30%) due to high overpotentials and methane's limited solubility in electrolytes, though it offers significant potential for decentralized and low‐emission applications.

**Electric field‐assisted activation**, a more recent innovation, provides an intriguing route to reduce activation barriers and enhance catalytic activity at lower temperatures by polarizing active sites or intermediates. This method has shown promising methane conversion rates and selectivity enhancements, especially in oxidative coupling and reforming reactions, with the added benefit of potentially minimizing coke formation. However, system‐level energy efficiencies are still underexplored, and scalability depends heavily on field uniformity and reactor design.

**Plasma‐assisted conversion** enables high methane activation rates and non‐oxidative coupling under mild bulk temperatures, but faces challenges with energy losses in plasma generation and limited product selectivity, making it better suited for niche or hybrid systems unless efficiency can be improved.

**Electrothermal methods** such as Joule and induction heating offer the highest energy efficiency (up to 95% for Joule heating in optimized systems) and better integration into existing reforming infrastructure, although they rely on high operational temperatures and robust materials.

From a scalability perspective, electrothermal methods are currently the most mature, while electrocatalytic systems show promise for modular, renewable‐powered setups. Environmentally, all three approaches can reduce GHG emissions when powered by low‐carbon electricity, with electrocatalysis and plasma pathways showing particular promise for CO_2_‐free product streams. A comparative summary is provided in Table 1 and 3 to help guide future research directions and process development strategies.

### 5.2 Environmental Impact and Carbon

The transition toward sustainable energy systems necessitate innovative approaches to address both the utilization and mitigation of methane emissions. Electrified methane conversion technologies have emerged as a promising pathway to achieve these goals, enabling efficient and scalable routes for methane valorization while using electricity from renewable sources. Electrocatalytic systems have demonstrated significant potential through advancements in catalyst design and reactor configurations. These systems enable methane activation under mild conditions, providing pathways to produce methanol, ethanol, and other hydrocarbons with high selectivity and efficiency. Similarly, plasma‐driven technologies leverage NTP to achieve high conversion rates, particularly in processes requiring lower temperatures and enhanced selectivity. Electro‐thermal methods, such as Joule and induction heating, present additional opportunities by integrating renewable electricity to power endothermic reactions, improving energy efficiency, and reducing carbon footprints.

Despite these advancements, several challenges remain to be addressed. Enhancing catalyst stability, improving reaction kinetics, and scaling up technologies for industrial applications are critical areas for future research. Further, optimizing energy consumption and integrating renewable energy sources into these processes will be essential to make them economically viable and sustainable.

Looking ahead, the integration of machine learning and artificial intelligence in catalyst design and process optimization holds immense potential to accelerate progress. Additionally, exploring hybrid systems that combine different electrified approaches may unlock new synergies and enhance overall performance. The deployment of modular and on‐site conversion units can further streamline methane utilization, especially in remote or offshore settings, reducing transportation costs and emissions.

In conclusion, electrified methane conversion technologies represent a transformative step toward a low‐carbon economy. By leveraging renewable energy and advanced catalytic systems, these methods offer a scalable and sustainable approach to methane utilization. Continued interdisciplinary research and collaboration will be key to overcoming existing barriers and realizing the full potential of these technologies for clean energy and chemical production.

## Conflict of Interest

The authors declare no conflict of interest.

## Author Contributions

**Alen Rupnik** wrote the main part of the manuscript and the first draft, **David Bajec** wrote the section on induction heating and revised the manuscript, **Igor Shlyapnikov** contributed with thermo‐catalytic section and improved the quality of the manuscript, **Miha Grilc** and **Gleb Veryasov** proposed the topic and the scope of this manuscript, helped structuring and improving the manuscript, **Blaž Likozar** provided supervision, resources, funding acquisition and project administration.
